# Executive function in children with neurodevelopmental conditions: a systematic review and meta-analysis

**DOI:** 10.1038/s41562-024-02000-9

**Published:** 2024-10-18

**Authors:** Ayesha K. Sadozai, Carter Sun, Eleni A. Demetriou, Amit Lampit, Martha Munro, Nina Perry, Kelsie A. Boulton, Adam J. Guastella

**Affiliations:** 1 Clinic for Autism and Neurodevelopment (CAN) Research, Brain and Mind Centre, Children’s Hospital Westmead Clinical School, Faculty of Medicine and Health, University of Sydney, Sydney, Australia; 2 Child Neurodevelopment and Mental Health Team, Brain and Mind Centre, University of Sydney, Sydney, Australia; 3 School of Biomedical Engineering, Faculty of Engineering, University of Sydney, Sydney, Australia; 4 Department of Psychiatry, University of Melbourne, Melbourne, Australia

**Keywords:** Autism spectrum disorders, Paediatric research, ADHD

## Abstract

Executive function (EF) delays are well documented in paediatric neurodevelopmental conditions (NDCs). There is no consensus about whether EF delay represents a transdiagnostic feature of NDCs. This systematic review and meta-analysis synthesized 180 studies reporting two or more NDC comparisons on EF, examined differences between NDCs, and the moderating effects of gender, age, publication year, DSM editions and assessment types. Studies using established EF measures across seven domains (attention, fluency, set-shifting, set-switching, response inhibition, planning and working memory) in participants under 18 were included. Summary effects were compared: (1) for all reported NDCs relative to control, (2) for each individual NDC relative to control and (3) between NDC groups. Results confirmed that EF delay was a transdiagnostic feature of neurodevelopmental delay, with a moderate effect size of delay across all NDCs (*g* = 0.56, 95% confidence interval (CI) 0.49–0.63) compared with control. This effect increased with comorbidities (*g* = 0.72, 95% CI 0.59–0.86), DSM-5 criteria and informant measures. Comparisons between NDCs revealed few differences: children with tic disorders (TD) showed smaller EF delays, children with attention-deficit/hyperactivity disorder (ADHD) showed larger delays in attention, response inhibition, planning and working memory compared with TD and specific learning disorders, while children with autism spectrum disorders showed greater delays on set-switching compared with ADHD. Findings support transdiagnostic models of neurodevelopment to further a developmentally sensitive science that can reveal how EF delays contribute to brain circuitry, symptom profiles and functioning, and ultimately support early interventions and outcomes for all children with NDCs.

## Abstract (web summary)

Sadozai et al. present a systematic review and meta-analysis of 180 studies on executive function delay in children with neurodevelopmental conditions such as autism spectrum disorder and attention-deficit/hyperactivity disorder.

## Main

Executive function (EF) is an umbrella term used to conceptualize a range of cognitive processes including planning, working memory, attention, inhibition, self-monitoring, self-regulation and initiation^1,2^. It encompasses a network of functional cognitive abilities that allow for real-world engagement, which are often refined in developmental periods^2,3^. Impairments in EF can include difficulties in sustaining attention, impulsivity and an inability to transition and flexibly manage multiple tasks^1,4–8^. Their presence may also signal divergence in brain development and can play a key role in both causal and maintaining factors in neurodevelopmental conditions (NDCs)^9,10^. Together, these processes are involved in a range of emotional, behavioural and social functions. Theoretical models over past decades propose that EF is comprised of core components that emerge early in development and are built upon over time, resulting in complex, higher-order cognitive abilities that are critical for daily functioning^11,12^. These models have been informed and supported by a substantial amount of empirical data^13,14^ and set the groundwork for this review. This review aims to investigate both higher-order and more foundational domains within the paediatric population. EF studies presents considerable debate on how EF domains overlap or present as separate faculties. Further, the literature attempts to capture these faculties through the use of various EF outcomes. Considering the body of literature in this area, this review will focus on attention, set-shifting, set-switching, fluency, planning, working memory and response inhibition. This also enables consideration of broader EF domains, such as attention^2^, where impairments have been consistently reported in NDCs such as attention-deficit/hyperactivity disorder (ADHD)^15^. Impairments in EF are believed to impact quality of life in children^16^ and can contribute to lifelong challenges^1,3^. These impairments are thought to be moderated by variables such as sex, type of EF measure and age^17^. For example, some studies have suggested that EF difficulties may increase during adolescence^18^, while others do not^19–21^. Overall, delays in EF could be considered to represent a broad transdiagnostic cognitive phenotype of NDCs. There has, however, been limited research examining this across NDCs.

EF impairments have traditionally been studied within specific NDCs^22,23^. Paediatric EF studies have generally focused on emerging core EF domains of inhibition, working memory and cognitive flexibility^11,24^. These domains are believed to serve foundational cognitive faculties that allow for the development of higher-order EF functions, such as planning and problem-solving^11^. Delays in each of these domains have been reported in children with NDCs in the first years of life^1,3^. So far, age-based effects have also been examined within disorders. For example, deficits in working memory are well documented in children with ADHD and specific learning disorders (SLD) across different developmental stages^25–27^.

Cross-disorder studies^23,28^ further show that there may be some differences in sub-domain EF outcomes based on the specific disorder phenotype. In support, some studies comparing children diagnosed with autism spectrum disorder (ASD) or ADHD suggest that children with ASD show marked delays in cognitive flexibility and planning, while children with ADHD have delays in inhibition and working memory^29–32^. Other studies^33^ have reported larger delays in response inhibition, cognitive flexibility/switching and working memory for children with ASD compared with those with ADHD^33^. Such comparisons are important to inform the understanding of causal and maintaining features that might differentiate clinical profiles of conditions. Further, they could be used to screen for diagnoses, to confirm co-occurring NDCs and to assess for clinical severity.

## EF measures in the paediatric population

Assessments of EF include both performance and informant-based measures. Performance EF tasks typically involve practical tasks of EF capacities (for example, errors on a computer-based response inhibition task; placing objects in an instructed order) and are purported to objectively tap into discrete EF domains. There is, however, a high degree of overlap between EF domains and their underlying neurobiology, raising questions about their functional independence^8^. As such, many measures of EF likely tap multiple domains. For example, tasks that measure set-shifting, such as the Wisconsin Card Sorting Test, may be impacted by a working memory component^34^. Other measures, such as informant-based measures, are proposed to possess greater ecological validity given their reliance on the reporting of observed everyday behaviours^1^. Informant-based measures typically describe how a child’s EF profile directly relates to their daily functioning, encompassing a greater number of observed functional deficits^17,22^. Such measures might have more ecological validity, but come at a potential cost of being less objective^25–27^. The debate between the ecological validity and objective reliability of these measures has been long-standing. Some authors have argued that performance-based measures may provide more objective scaling of EF performance, while others have pointed to the utility of informant and self-report measures to predict functioning outcomes; nonetheless, each provides unique clinical utility for a range of paediatric contexts^35–37^.

## Study aims

There is a lack of research examining EF as a transdiagnostic phenotype in paediatric NDCs. This meta-analysis aims to synthesize the existing literature of EF in NDCs. It reviews seven EF constructs identified in paediatric literature (attention, fluency, set-shifting, set-switching, response inhibition, planning and working memory). Given that EF is a component of cognition across neurodevelopment, it is critical to evaluate its discriminating and/or shared profile across NDCs. This meta-analysis aimed to review studies that tested EF measures across two or more NDCs in children with or without a control comparison. Further, this study aimed to consider how the type of assessment measure (informant or performance-based), DSM criteria and demographic factors (age and gender) influenced the results of the primary analysis. We predicted that:

1. NDCs would be associated with significant delays across EF domains, when compared with controls.
2. Effect size of delay would be moderated by the type of assessment and reported sex, such that informant-based measures and a higher percentage of males captured in studies would be associated with larger effect sizes.
3. Comparisons of EF profiles between NDCs would show differences in the severity of delay overall and for EF sub-domains.

## Results

### Primary outcome: neurodevelopmental groups and controls

**Fig. 1:**
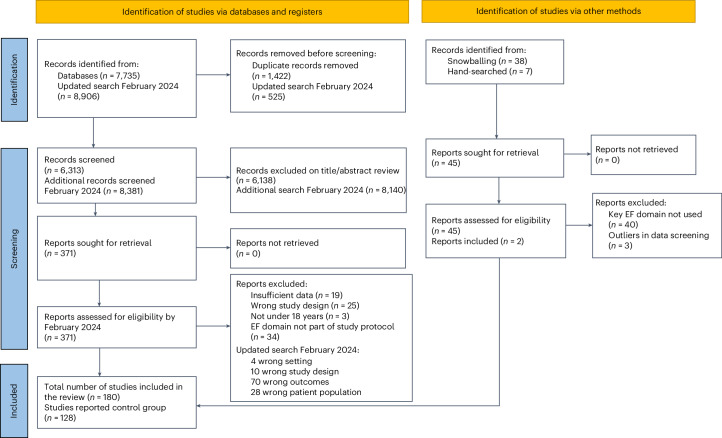
The PRISMA diagram of this systematic review. The retrieval processes are illustrated, covering the stages of identification, screening and inclusion of studies. Note that the use of *n* indicates the number of studies included at each stage.

**Fig. 2:**
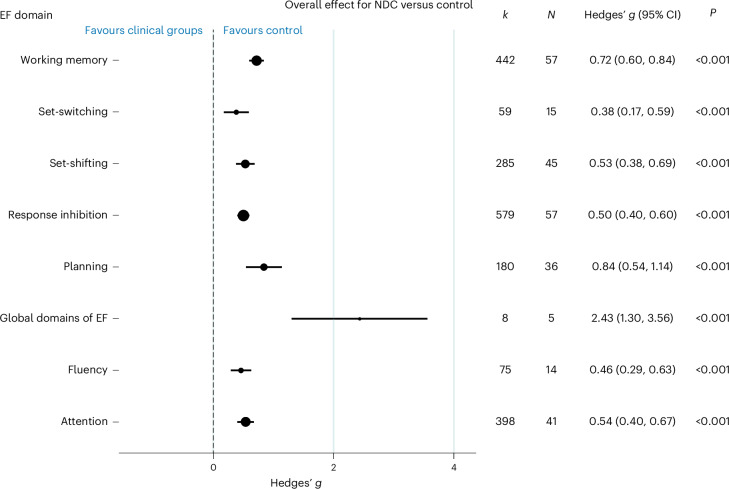
Forest plot summaries overall effects (Hedges’ *g*) for EF in NDCs versus controls. Here *N* refers to the number of studies across EF comparisons made within the meta-analysis, and *k* refers to the number of outcome measures reported. Mean effects and their 95% CI are presented by the central black circle and the horizontal line. The size of the black circle reflects the number of studies included in each comparison. The dashed line at *g* = 0 indicates no effect, while the *P* values show whether the effect was statistically significant. The *P* values reported from the random-effects models are two-sided. Source data

An overall meta-analysis comparing eligible studies that compared two or more NDCs with controls was completed (see Fig. 1 for the literature search process and Supplementary Table 3 for study characteristics). To facilitate the analysis and accurate interpretation of our findings, we systematically reported Hedges’ *g* effect size estimates (*g*), with 95% confidence interval (CI) when appropriate, and reported variances (as detailed in Methods). The neurodevelopmental groups showed significant impairment in their overall EF (*N* = 114 studies, *k* = 2031 outcomes, *g* = 0.56, 95% CI = 0.49 to 0.63, *P* < 0.001, *τ*^2^ = 0.14, prediction interval −0.17 to 1.29). These effect sizes overall were statistically significant for both performance (*N* = 110 studies, *k* = 1911 outcomes, *g* = 0.51, *P* < 0.001) and informant-based measures (*N* = 14 studies, *k* = 120 outcomes, *g* = 1.49, *P* < 0.001) and across all domains of EF (Fig. 2).

Funnel plot asymmetry was detected with Egger’s test, indicating a possible small-study effect (*β* = −0.31, *P* < 0.001). A trim-and-fill analysis did not find any missing studies (Supplementary Fig. 1). Inspection of the funnel plot revealed that asymmetry was driven by the informant measurement outcomes (with *g* > 2). Five studies were removed as they contributed to plot asymmetry (Supplementary Table 4). The overall effect stayed the same (*g* = 0.56, 95% CI = 0.49 to 0.63, *P* < 0.001, *τ*^2^ = 0.14, prediction interval −0.17 to 1.29).

**Fig. 3:**
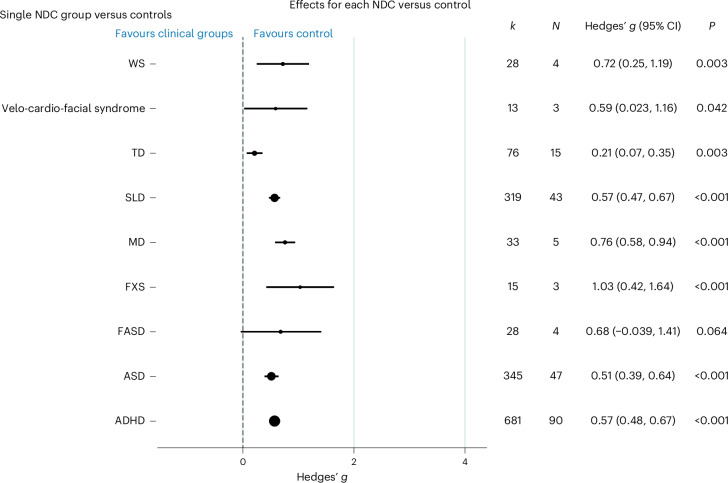
Forest plot summaries overall effects (Hedges’ *g*) for each single NDC versus control. Here *N* refers to the number of studies reported, and *k* refers to the number of outcome measures reported. Mean effects and their 95% CI are presented by the central black circle and the horizontal line. The size of the black circle reflects the number of studies included in each comparison. The dashed line at *g* = 0 indicates no effect, while the *P* values show whether the effect was statistically significant. The *P* values reported from the random-effects models are two-sided. FASD, fetal alcohol spectrum disorder; velo-cardio-facial syndrome (including 22q11DS), WS, fragile X syndrome (FXS), motor disorders (MD). Other group comparisons, including DS, neurofibromatosis-1, intellectual disability, Turner syndrome, communication disorders (speech, language and specific language impairments), Prader–Willi syndrome, developmental disability and prenatal alcohol exposure, were not conducted (*N* ≤ 2). Source data

A meta-analysis was conducted comparing paediatric NDCs and their control group (Fig. 3). Significant delays in overall EF were observed for all neurodevelopmental groups, except FASD, and across all sub-domains (Fig. 2). Effect sizes were moderate to large, except for TD where a small effect size was observed. For FASD, the sample collected was imprecise with a small number of studies (*N* = 4 studies) to contribute to this observation. For those conditions with the greatest number of studies, effect sizes were moderate in size (ADHD, ASD and SLD), suggesting similar delays. Analysis was then conducted between those studies that specifically recruited comorbid NDC groups of children (for example, children diagnosed with both ASD and SLD) against those studies that only required a single NDC diagnosis for inclusion. Studies focused on recruiting comorbid NDCs showed a larger effect size of EF delay (*N* = 58 studies, *k* = 427 outcomes, *g* = 0.72, 95% CI = 0.59 to 0.86, *P* < 0.001) (as shown in Supplementary Table 5) against studies that only required diagnosis of a single NDC (*N* = 113 studies, *k* = 1601 outcomes, *g* = 0.53, 95% CI = 0.46 to 0.60, *P* < 0.001).

### Moderator analysis

There was a small, though significant, moderator effect of reported sex, based on the percentage of males (*N* = 92 studies, *k* = 1746 outcomes, *β* = −0.003, 95% CI = −0.005 to −0.001, *P* = 0.016, *Q*_(1)_ = 5.84, *R*^2^ = 1.33%). The higher the percentage of males, the smaller was the effect size of NDC compared with control, but this accounted for a negligible degree of variance. The moderator effect of age was not significant (*N* = 111 studies, *k* = 1995 outcomes, *g* = −0.027, 95% CI = −0.076 to 0.021, *P* = 0.267, *Q*_(1)_ = 1.23, *R*^2^ = 0.82%).

The effect of DSM edition used in assessments was significant (*N* = 84 studies, *k* = 1,452 outcomes, *P* < 0.001, *Q*_(2)_ = 75.25, *R*^2^ = 22.92). DSM-III (*g* = 0.49, 95% CI = 0.31 to 0.66, *P* < 0.001) had a similar effect size to DSM-IV (*g* = 0.47, 95% CI = 0.39 to 055, *P* < 0.001). However, effect sizes increased significantly with the use of the DSM-5 edition (*g* = 0.92, 95% CI = 0.81 to 1.03, *P* < 0.001). This effect held when examining only performance-based measures *(N* = 80 studies, *k* = 1363 outcomes, *P* < 0.001, *Q*_(2)_ = 19.45, *R*^2^ = 25.38%), with the use of DSM-III (*g* = 0.43, 95% CI = 0.27 to 0.59, *P* < 0.001), DSM-IV (*g* = 0.46, 95% CI = 0.39 to 0.53, *P* < 0.001) and DSM-5 (*g* = 0.73, 95% CI = 0.61 to 0.85, *P* < 0.001), when each single disorder (TD, ASD, ADHD, SLD and developmental coordination disorder (DCD) was separately removed from the analysis, and when comorbid NDCs were removed from analysis. Informant-based measures did not show significant differences (*N* = 11 studies, *k* = 89 outcomes, *P* = 0.592, *Q*_(1)_ = 0.29, *R*^2^ = 0%). Publication year was not significantly different (*N* = 111 studies, *k* = 1964 outcomes, *g* = 0.001, 95% CI = −0.006 to 0.007, *P* = 0.868, *Q*_(1)_ = 0.028, *R*^2^ = 0.17%).

Moderator analyses also revealed that there were significant moderator effects based on the type of EF assessment measure (*N* = 113 studies, *k* = 2028 outcomes, *Q*_(1*)*_ = 717.47, *P* < 0.001, *R*^2^ = 19.08%). Effect sizes for performance measures (*g* = 0.49, 95% CI = 0.43 to 0.56, *P* < 0.001) were significantly smaller compared with informant measures (*g* = 1.47, 95% CI = 1.38 to 1.56, *P* < 0.001).

### EF profiles in specific NDCs

**Fig. 4:**
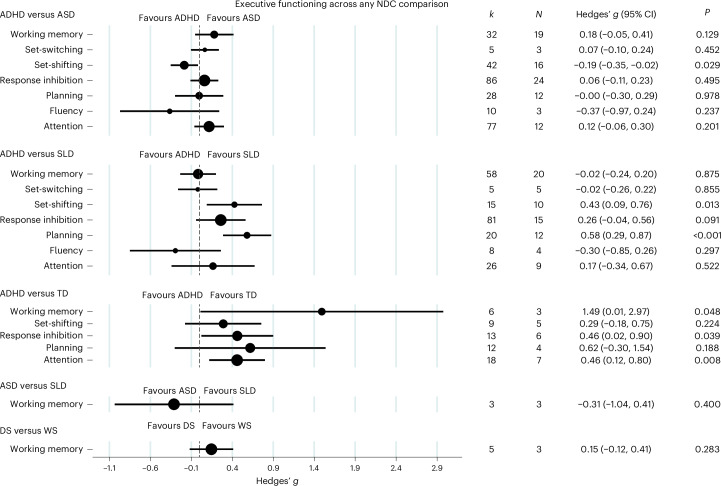
Effect sizes for seven areas of EF across NDC comparisons. Mean effects and their 95% CI are presented by the central black circle and the horizontal line. The size of the black circle reflects the number of studies included in each comparison. The dashed line at *g* = 0 indicates no effect, while the *P* values show whether the effect was statistically significant. The *P* values reported from the random-effects models are two-sided. Source data

The next set of analyses examined whether the observed delay found on EF measures differed according to the neurodevelopmental diagnosis (see Supplementary Table 6 for details). To address this question, we conducted a series of analyses between each NDC using studies that provided enough power (*N* ≥ 3 studies). This was informed by the presence of underlying heterogeneity as well as a sufficient number and balance of studies within subgroups to allow for cross-condition analyses to take place within each cell (Fig. 4 and Supplementary Table 8). In the majority of cases, there were not sufficient data to explore both performance and informant outcomes separately (results from these analyses where available are provided in Supplementary Fig. 2).

#### ADHD and ASD

Studies comparing ADHD and ASD showed that there were no significant differences on overall EF measures between conditions (*N* = 39 studies, *k* = 284 outcomes, *g* = 0.076, 95% CI = −0.026 to 0.18, *P* = 0.146, *τ*^2^ = 0.088, prediction interval −0.52 to 0.67). The sub-domain comparisons, however, revealed that children with ADHD performed better on measures of set-shifting compared with children with ASD *(N* = 16 studies, *k* = 42 outcomes, *g* = −0.19, 95% CI = −0.35 to −0. 019, *P* = 0.029).

#### ADHD and TD

There was a significant effect for overall EF domain differences (*N* = 12 studies, *k* = 60 outcomes, *g* = 0.52, 95% CI = 0.22 to 0.82, *P* < 0.001, *τ*^2^ = 0.26, prediction interval −0.53 to 1.56), suggesting that the ADHD group showed poorer performance than the TD group. Sub-domain analyses for individual EF domains showed differences in attention (*N* = 7 studies, *k* = 18 outcomes, *g* = 0.46, 95% CI = 0.12 to 0.80, *P* = 0.008), response inhibition *(N* = 6 studies, *k* = 13 outcomes, *g* = 0.46, 95% CI = 0.023 to 0.9, *P* = 0.039) and working memory (*N* = 3 studies, *k* = 6 outcomes, *g* = 1.49, 95% CI = 0.013 to 2.98, *P* = 0.048). Children with TD performed better than children with ADHD on these domains.

#### ADHD and SLD

Studies comparing ADHD and SLD showed no significant results for a combined effect on EF measures (*N* = 33 studies, *k* = 213 outcomes, *g* = 0.099, 95% CI = −0.066 to 0.26, *P* = 0.238, *τ*^2^ = 0.20, prediction interval −0.80 to 1.0). The sub-domain analyses revealed that the SLD group performed better on tests of planning (*N* = 12 studies, *k* = 20 outcomes, *g* = 0.58, 95% CI = 0.29 to 0.88, *P* < 0.001) and set-shifting (*N* = 10 studies, *k* = 15 outcomes, *g* = 0.43, 95% CI = 0.09 to 0.76, *P* = 0.013).

#### ADHD and DCD

Findings from studies comparing ADHD and DCD suggested no overall differences between groups (*N* = 3 studies, *k* = 18 outcomes, *g* = −0.37, 95% CI = −0.94 to 0.20, *P* = 0.203, *τ*^2^ = 0.20, prediction interval −1.42 to 0.68). Sub-domain analyses were not conducted owing to an insufficient number of studies (*N* < 3 studies).

#### ASD and SLD

A review of studies comparing children with ASD and SLD revealed no differences between groups (*N* = 4 studies, *k* = 9 outcomes, *g* = 0.046, 95% CI = −0.7 to 0.79, *P* = 0.904, *τ*^2^ = 0.53, prediction interval −1.57 to 1.66). No significant results were found when conducting sub-domain analyses. We note that this sub-domain comparison did not include tests of set-shifting.

#### Down syndrome (DS) and Williams syndrome (WS)

Studies reporting EF measures between DS and WS suggested no overall significant differences between groups (*N* = 4 studies, *k* = 32 outcomes, *g* = −0.035, 95% CI = −0.40 to 0.32, *P* = 0.846, *τ*^2^ = 0.12, prediction interval −0.80 to 0.73). There were not sufficient studies to explore sub-domain differences.

## Discussion

Our results suggest that EF delay is a transdiagnostic feature of paediatric NDCs, with an overall moderate effect size of delay compared with control. Comparisons between specific NDCs showed few differences on sub-domains. Children diagnosed with TD showed small EF delays overall, while children with ADHD showed larger delays on attention, response inhibition and planning on some comparisons to children with TD and SLDs. Children with ASD showed poorer set-shifting when compared with children with ADHD. We then conducted moderator analyses to understand factors that might contribute to larger effect sizes. Moderator analysis showed that EF delay increased with the percentage of females in each study, but the meaningful contribution of reported sex was marginal. There was no influence of age. While there was no moderating effect of publication year, EF delay was substantially increased with the use of the DSM-5 criteria compared with previous DSM editions. The effect of DSM-5 appeared stable regardless of specific NDCs or measures. Informant-based measures of EF, relative to performance-based measures, showed much larger EF delays. Results suggest EF delay is a transdiagnostic phenotype of NDCs and highlight an urgent need for research that captures data across NDCs. Such data are needed to inform a broader transdiagnostic science of neurodevelopment^38^ and to inform how delays impact circuits, symptoms and functioning across child development.

The findings of this study suggest a broad EF delay across all NDCs, with little evidence that specific NDCs were associated with greater overall EF delays. The executive system is believed to be one of the most recently evolved brain systems that operates as a control centre, managing other cognitive abilities (for example, attention, learning, social development and memory)^1,3^. It facilitates flexibility and adaptability^39^ to novel and complex situations. While its circuitry is facilitated by the prefrontal cortex, it is highly connected and reliant on other brain circuitry^6^. It follows then that any divergence in brain development in EF systems is likely to impact this interconnected system.

Research has also shown how genetic, neurochemical and environmental^40,41^ factors linked to NDCs, as well as critical periods of neurodevelopment, may all influence EF maturation. Importantly, the effect of EF delay in comorbid recruited studies was larger than for studies that only required single NDCs for recruitment. Comorbidity has long been associated with poorer outcomes within reviews of specific NDCs^22^. Such findings suggest that EF delay may provide a sensitive marker to differentiate NDC complexity and progression. A transdiagnostic NDC research framework^38^ is now needed to further evaluate modifiers of EF across child development that can better incorporate the genetic and environmental contributors to these effects across development and functioning^42,43^.

Only one difference emerged between specific NDC conditions on the overall EF outcome. Results showed that children with TD had a smaller EF delay compared with children with ADHD, which was consistent with a small effect size of delay for children with TD when compared with control populations. Past studies have suggested that children with TD may experience greater impacts from circuits that drive motor function in combination with EF delays^44^, and this circuitry may additionally impact functioning. Further research is required to understand such interactions between motor and EF performance and the circuits that underpin outcomes in child development.

While EF delay was largely transdiagnostic, some sub-domain comparison showed differences between NDCs. Children with ADHD showed greater delay on attention, response inhibition and working memory, compared with children with TDs, and planning and set-shifting compared with children with SLDs. Attention disruption is central to the diagnostic criteria of ADHD^45^ and the results reinforce this on both informant and performance-based measures. Results offer some support to the specificity of attention-based models of ADHD^15,46,47^ that highlight brain regions (such as the dorsal anterior cingular cortex) involving selective and sustained attention. According to the supervisory attentional system^46^, delayed attention can cause challenges in disengaging from habitual behaviour and exerting novel responses, which, in turn, implicates response inhibition and set-shifting^48^. While such models allude to the unique role attention holds within the EF network and its multi-layered nature^2,49^, others take a multifactorial approach and highlight the trajectory EF domains take from simple to complex EF networks^6,11^. These models emphasize the interconnected relationship attention has with other EF domain areas as EF networks become more mature^50^. In regards to other conditions, children with ASD were more delayed on tests of set-shifting (also known as concept formation) compared with their ADHD counterparts^22,51^. This delay has been linked to the phenotype of rigidity and repetitive behaviours, as well as difficulties in processing stimuli and social information in complex environments^52,53^. Taken together, these findings inform our understanding that while NDCs share an overall transdiagnostic EF profile^54,55^, nuanced atypicalities could lead to distinct cognitive profiles^22,33,43,51,56^.

Interestingly, DSM edition was a significant moderator, suggesting that the effect size of EF delay for NDCs has almost doubled in size since the introduction and use of the DSM-5. These findings could not be accounted for by any one specific diagnosis, since the removal of a single diagnosis, or removal of comorbid NDCs, from this analysis did not alter the significance. The effect was also found when limiting analysis to only performance or informant reports, so it is unlikely to be due to EF measurement changes. It is also unlikely to be accounted for a real increase in EF delay over the last 40 years, since publication year was not a significant moderator. There has, however, been a gradual increase in the percentage of studies focusing on ASD and a reduction in the percentage of studies focusing on TDs (Supplementary Table 7). Furthermore, there have been important changes in criteria that permit both ASD and ADHD as comorbid conditions. We cannot rule out that a changing focus of NDC research may contribute to this effect. Further prospective research is required to determine whether this effect of DMS-5 assessment criteria is a direct consequence of the changing criteria or changing research priorities since the release of DSM-5.

Assessment type had a significant contribution to the overall effect. Findings of larger EF delays when using performance versus informant-based measures have also been supported by previous literature^22,37^. The administration of performance-based measures evaluates EF in cumulative scores that tap into accuracy of individual cognitive domains, whereas informant-based measures are often based on reporting observed challenges on rating scales, which are correlated with functionality or real-world behavioural performance of the individual. A large number of studies utilize performance-based measures, which allows for EF domains to be siloed and more precisely and objectively assessed^37^. However, some research has shown that informant EF measures may be better at differentiating those with a clinical diagnosis as it captures the degree and type of impairment in everyday life^22^. Taken together, these findings suggest that research is needed to delineate the use of informant and performance-based measures across development and their utility to understand when informant or performance-based measures are best utilized, with some research suggesting the increased value of using both^37^.

## Limitations

Although many studies control for IQ, we did not include IQ as a primary covariate in our analysis given the overlap between IQ and EF domains. We also note the low number of studies used to identify EF patterns in lesser investigated NDCs. This meta-analysis is limited by the existing literature and the absence of broad investigations of neurodevelopment that incorporates many between-disorder comparisons. This study highlights the urgent need for future work to address these gaps in transdiagnostic developmental research (Supplementary Table 8) where greater focus may be placed on other non-diagnostic contributors to EF delay. The results here show that when a cross-disorder evaluation of EF domains is applied, it leads to a more integrated and nuanced understanding of the EF impairment across sub-domains and overall in child development. Considering that this review was limited by the number of studies captured within a particular NDC, we note lack of generalizability to lesser investigated NDCs and lack of generalizability to lesser utilized experimental methods (that is, longitudinal studies). Finally, we acknowledge that many of the included studies did not control for comorbidity and the changing criteria in DSM iterations is a limitation for diagnostic specific comparisons given the changes to discrete diagnostic classification over time.

## Conclusion

The conclusions drawn here demonstrate that EF delay is a transdiagnostic feature of paediatric NDCs that increases in severity with greater diagnostic complexity. Future research should target transdiagnostic and distinct EF profiles across the developmental trajectory with a focus on determinants of variance in the EF phenotype, such as its neurobiological underpinnings (for example, functional brain connectivity and neurotransmission) and environmental factors that moderate outcomes across age. Delays in EF may also provide useful and stable transdiagnostic markers for the identification of neurodevelopmental delays that can be used relatively early in life. Subtle differences observed here between NDCs may also further offer potential to develop precision medicine approaches for specifying the circuits underpinning the development of different NDCs, leading to targeted supports for learning, cognition and daily living.

## Methods

This review adheres to the Preferred Reporting Items for Systematic Reviews and Meta-analyses (PRISMA) guidelines^57,58^. The review was prospectively registered with PROSPERO (CRD42020210785).

### Study selection

The review included peer-reviewed studies in any language published from 1980 to 19 February 2024 (see Fig. 1 for the PRISMA diagram). Cross-sectional and longitudinal cohort studies were considered if they included children under 18 years of age and reported on at least two NDC comparison groups (see Supplementary Table 1 for search strategy). NDCs were identified if they were listed within the DSM-5 and assessed with reliable diagnostic measures.

### Search strategy and study variables

The literature search was conducted through MEDLINE, Embase and PsycINFO databases using detailed search criteria of EF measures (that is, ‘BRIEF’, ‘Wisconsin Card Sorting Test’, ‘Go-No-Go task’ and ‘Stroop test’) and a full range of paediatric NDCs (for example, ‘ASD’, ‘SLD’, ‘ADHD’ and ‘TD’). Terms used to describe NDCs in previous DSM editions were also included in the search strategy, as were NDCs identified in The SAGE Handbook of Developmental Disorders^59^. See Supplementary Tables 1 and 2 for the search strategy and more detailed descriptions of included EF measures and NDCs. Each NDC comparison then needed sufficient data (at least three studies) to be included for further analysis. The search strategy captured information such as whether the diagnosis was made through a clinical interview, DSM criteria, standardized measures or other modes of classification. In addition, studies must have included established outcome measures looking at either informant measures or performance measures of EF (Supplementary Table 1).

The authors (A.K.S., C.S., N.P. and M.M.) screened for initial eligibility based on title and abstract, and then for the full-text screenings using Covidence (Extraction 1)^60^. An independent reviewer addressed any disagreements (E.A.D.). The inter-rater reliability, kappa value, was 0.87, indicating high agreement between reviewers^61^.

In data extraction, EF measure outcomes (that is, commissions or omissions errors in a task like Go-No-Go) reported in each comparison group were extracted as mean values and standard deviation scores at a single time point (or baseline results were extracted in the case of longitudinal studies). For studies reporting multiple measures derived from psychometric tests, experimental tasks and/or self/informant measures, each outcome measure was considered separately. Where there were missing data, efforts were made to contact authors regarding missing data by email at least once; however, no author was able to address these requests. In addition, all study authors were contacted for unpublished data to mitigate ‘the file drawer effect’. One author was able to address this request and their data are included in the results (Supplementary Table 3). The data extraction process was completed using a customized Excel spreadsheet (version 2404).

### Quality assessment

Quality review of studies was completed by assessors (A.K.S., C.S., M.M. and N.P.) using the Checklist for Cross-Sectional and Cohort Studies within the JBI Critical Appraisal Tools. Studies were based on the JBI critical appraisal tool with 80% of ratings double coded. Agreement between raters (kappa value) was 0.97, indicating high agreement between reviewers^61^.

### Data items

Group-level summary data (for example, sample size, means and standard deviations of each group) from each was extracted for all measures reporting outcomes for EF. When studies reported data from multiple measures or subgroups, all eligible data were extracted. When studies reported outcomes across several manuscripts, data were combined into a single study to avoid double-counting of studies. All meta-analyses were conducted with the metafor (version 3.8-1), meta (version 6.5-0), dplyr (version 1.1.2), readxl (version 1.4.3) packages in RStudio (2023.06.1 Build 524) and R (version 4.3.3) using multivariate models in order to account for non-independence among effect sizes within studies. The unit of analysis utilized within this model was the standardized mean difference (calculated as Hedges’ *g*) on each measure between each comparison group. When making NDC versus control group comparisons, a positive effect size indicated that the control group performed better than the NDC group. The data analysis was planned a priori and was completed in four stages. The initial analysis combined all EF outcomes to assess the overall EF effect size across NDCs when compared with controls. Publication bias was assessed using Egger’s test and trim-and-fill methods and illustrated in funnel plots. The second analysis examined subgroup comparison of the individual EF domains. The third analysis examined the estimated effect size of difference for each NDC compared with control conditions. The next step involved examining between study variability and moderator impact for overall EF and individual EF domains. This included ‘type of measure’, ‘gender’, ‘age’, ‘DSM edition’ and ‘year of publication’, which were assessed as covariates in meta-regression analyses. The final step involved the analyses of individual EF domains for each of the NDC comparison groups (that is, ASD versus ADHD and so on). By convention, effect sizes (Hedges’ *g*) with 95% CI are described as small (⩽0.30), moderate (>0.30 and <0.60) and large (⩾0.60). Heterogeneity (that is, variance between studies) was quantified using *τ*^*2*^. The variance explained by the moderators in the meta-regression model was quantified as *R*^2^ and formally tested using Cochran’s *Q*.

### Reporting summary

Further information on research design is available in the Nature Portfolio Reporting Summary linked to this article.

## Supplementary information

Supplementary Information Supplementary Figs. 1 and 2 and Tables 1–8.

Reporting Summary

Supplementary Data 1 Statistical analysis results from metafor package for Supplementary Fig. 1a,b. Supplementary Fig. 2. Effect sizes for seven areas of EF across neurodevelopmental comparisons with (a) performance only and (b) informant only measures. Mean effects and their 95% confidence intervals are presented by the central black circle and the horizontal line. The size of the black circle reflects the number of studies included in each comparison. The dashed line at *g* = 0 indicates no effect, while the *P* values show whether the effect was statistically significant. The *P* values reported from the random-effects models are two-sided.

## Source data

Source Data Fig. 2 Statistical source data.

Source Data Fig. 3 Statistical source data.

Source Data Fig. 4 Statistical source data.

## Data Availability

The data used to undertake this systematic review and meta-analysis are freely available (https://github.com/CarterSunUSYD/Transdiagnostic_EF_meta.git). Source data are provided with this paper.

## Code availability

The R codes used for the meta-analysis are freely available (https://github.com/CarterSunUSYD/Transdiagnostic_EF_meta.git).

## References

[1] Goldstein, S. & Naglieri, J. A. (eds) *Handbook of Executive Functioning* (Springer, 2014).

[2] Lezak, M. D., Howieson, D. B., Bigler, E. D. & Tranel, D. *Neuropsychological Assessment* 5th edn (Oxford Univ. Press, 2012).

[3] Hoskyn, M., Iarocci, G. & Young, A. R. *Executive Functions in Children’s Everyday Lives: A Handbook for Professionals in Applied Psychology* (Oxford Univ. Press, 2017).

[4] Anderson, P. J. & Reidy, N. Assessing executive function in preschoolers. *Neuropsychol. Rev.* **22**, 345–360 (2012). 23109046 10.1007/s11065-012-9220-3

[5] Bell, M. A. & Cuevas, K. in *Executive Function in Preschool-Age Children: Integrating Measurement, Neurodevelopment, and Translational Research* 157–179 (American Psychological Association, 2016).

[6] Diamond, A. Executive functions. *Annu. Rev. Psychol.* **64**, 135–168 (2013). 23020641 10.1146/annurev-psych-113011-143750 PMC4084861

[7] Hughes, C. & Graham, A. Measuring executive functions in childhood: problems and solutions? *Child Adolesc. Ment. Health* **7**, 131–142 (2002).

[8] Hunter, S. J. & Sparrow, E. P. (eds) *Executive Function and Dysfunction: Identification, Assessment and Treatment* (Cambridge Univ. Press, 2012).

[9] Miyake, A. & Friedman, N. P. The nature and organization of individual differences in executive functions: four general conclusions. *Curr. Dir. Psychol. Sci.* **21**, 8–14 (2012). 22773897 10.1177/0963721411429458 PMC3388901

[10] Capilla, A. et al. [Emergence and brain development of executive functions]. *Actas Esp. Psiquiatr.* **32**, 377–386 (2004). 15529228

[11] Miyake, A. et al. The unity and diversity of executive functions and their contributions to complex ‘frontal lobe’ tasks: a latent variable analysis. *Cogn. Psychol.* **41**, 49–100 (2000). 10945922 10.1006/cogp.1999.0734

[12] Diamond, A. Evidence of robust recognition memory early in life even when assessed by reaching behavior. *J. Exp. Child Psychol.* **59**, 419–456 (1995). 7622987 10.1006/jecp.1995.1020

[13] Snyder, H. R., Miyake, A. & Hankin, B. L. Advancing understanding of executive function impairments and psychopathology: bridging the gap between clinical and cognitive approaches. *Front. Psychol.* **6**, 328 (2015). 25859234 10.3389/fpsyg.2015.00328 PMC4374537

[14] Baggetta, P. & Alexander, P. A. Conceptualization and operationalization of executive function. *Mind Brain Educ.* **10**, 10–33 (2016).

[15] Mueller, A., Hong, D. S., Shepard, S. & Moore, T. Linking ADHD to the neural circuitry of attention. *Trends Cogn. Sci.* **21**, 474–488 (2017). 28483638 10.1016/j.tics.2017.03.009 PMC5497785

[16] de Vries, M. & Geurts, H. Influence of autism traits and executive functioning on quality of life in children with an autism spectrum disorder. *J. Autism Dev. Disord.* **45**, 2734–2743 (2015). 25835211 10.1007/s10803-015-2438-1 PMC4553152

[17] Demetriou, E. A., DeMayo, M. M. & Guastella, A. J. Executive function in autism spectrum disorder: history, theoretical models, empirical findings, and potential as an endophenotype. *Front. Psychiatry* **10**, 753 (2019). 31780959 10.3389/fpsyt.2019.00753 PMC6859507

[18] Skogli, E. W., Andersen, P. N., Hovik, K. T. & Øie, M. Development of hot and cold executive function in boys and girls with ADHD: a 2-year longitudinal study. *J. Atten. Disord.* **21**, 305–315 (2017). 24626329 10.1177/1087054714524984

[19] Biederman, J. et al. Stability of executive function deficits in girls with ADHD: a prospective longitudinal followup study into adolescence. *Dev. Neuropsychol.* **33**, 44–61 (2007). 10.1080/87565640701729755 18443969

[20] Anderson, V. A., Anderson, P., Northam, E., Jacobs, R. & Catroppa, C. Development of executive functions through late childhood and adolescence in an Australian sample. *Dev. Neuropsychol.* **20**, 385–406 (2001). 11827095 10.1207/S15326942DN2001_5

[21] Boelema, S. R. et al. Executive functioning shows differential maturation from early to late adolescence: longitudinal findings from a TRAILS study. *Neuropsychology* **28**, 177 (2014). 24364395 10.1037/neu0000049

[22] Demetriou, E. A. et al. Autism spectrum disorders: a meta-analysis of executive function. *Mol. Psychiatry* **23**, 1198–1204 (2018). 28439105 10.1038/mp.2017.75 PMC5984099

[23] Kingdon, D., Cardoso, C. & McGrath, J. J. Research review: executive function deficits in fetal alcohol spectrum disorders and attention-deficit/hyperactivity disorder—a meta-analysis. *J. Child Psychol. Psychiatry* **57**, 116–131 (2016). 26251262 10.1111/jcpp.12451 PMC5760222

[24] Lehto, J. E., Juujärvi, P., Kooistra, L. & Pulkkinen, L. Dimensions of executive functioning: evidence from children. *Br. J. Dev. Psychol.* **21**, 59–80 (2003).

[25] Isquith, P. & Gioia, G. *Behavior Rating Inventory of Executive Function*™ *BRIEF* (Psychological Assessment Resources, 2002).

[26] Isquith, P. & Gioia, G. *Behavior Rating Inventory of Executive Function*®—*Preschool Version* (Psychological Assessment Resources, 2003).

[27] Gioia, G. A., Espy, K. A. & Isquith, P. K. *BRIEF-P: Behavior Rating Inventory of Executive Function*—*Preschool Version* (Psychological Assessment Resources, 2003).

[28] Jang, J. et al. Rates of comorbid symptoms in children with ASD, ADHD, and comorbid ASD and ADHD. *Res. Dev. Disabil.* **34**, 2369–2378 (2013). 23708709 10.1016/j.ridd.2013.04.021

[29] Bramham, J. et al. Evaluation of group cognitive behavioral therapy for adults with ADHD. *J. Atten. Disord.* **12**, 434–441 (2009). 18310557 10.1177/1087054708314596

[30] Geurts, H. M., Verté, S., Oosterlaan, J., Roeyers, H. & Sergeant, J. A. How specific are executive functioning deficits in attention deficit hyperactivity disorder and autism? *J. Child Psychol. Psychiatry* **45**, 836–854 (2004). 15056314 10.1111/j.1469-7610.2004.00276.x

[31] Sinzig, J., Bruning, N., Morsch, D. & Lehmkuhl, G. Attention profiles in autistic children with and without comorbid hyperactivity and attention problems. *Acta Neuropsychiatr.* **20**, 207–215 (2008). 25385656 10.1111/j.1601-5215.2008.00292.x

[32] Martinussen, R., Hayden, J., Hogg-Johnson, S. & Tannock, R. A meta-analysis of working memory impairments in children with attention-deficit/hyperactivity disorder. *J. Am. Acad. Child Adolesc. Psychiatry* **44**, 377–384 (2005). 15782085 10.1097/01.chi.0000153228.72591.73

[33] Corbett, B. A., Constantine, L. J., Hendren, R., Rocke, D. & Ozonoff, S. Examining executive functioning in children with autism spectrum disorder, attention deficit hyperactivity disorder and typical development. *Psychiatry Res.* **166**, 210–222 (2009). 19285351 10.1016/j.psychres.2008.02.005 PMC2683039

[34] Kolakowsky-Hayner, S. A. in *Encyclopedia of Clinical Neuropsychology* (eds Kreutzer, J. S. et al.) 2719–2720 (Springer New York, 2011).

[35] Krieger, V. & Amador-Campos, J. A. Assessment of executive function in ADHD adolescents: contribution of performance tests and rating scales. *Child Neuropsychol.* **24**, 1063–1087 (2018). 29041835 10.1080/09297049.2017.1386781

[36] Isquith, P. K., Roth, R. M. & Gioia, G. Contribution of rating scales to the assessment of executive functions. *Appl. Neuropsychol. Child* **2**, 125–132 (2013). 23442015 10.1080/21622965.2013.748389

[37] Toplak, M. E., West, R. F. & Stanovich, K. E. Practitioner review: do performance-based measures and ratings of executive function assess the same construct? *J. Child Psychol. Psychiatry* **54**, 131–143 (2013). 23057693 10.1111/jcpp.12001

[38] Boulton, K. A. et al. A national harmonised data collection network for neurodevelopmental disorders: a transdiagnostic assessment protocol for neurodevelopment, mental health, functioning and well‐being. *JCPP Adv.* **1**, e12048 (2021). 37431407 10.1002/jcv2.12048 PMC10242941

[39] Van der Linden, M., Meulemans, T., Marczewski, P. & Collette, F. The relationships between episodic memory, working memory, and executive functions: the contribution of the prefrontal cortex. *Psychol. Belg.* **40**, 275 (2000).

[40] Rhoades, B. L., Greenberg, M. T., Lanza, S. T. & Blair, C. Demographic and familial predictors of early executive function development: contribution of a person-centered perspective. *J. Exp. Child Psychol.* **108**, 638–662 (2011). 20828709 10.1016/j.jecp.2010.08.004 PMC3016464

[41] Chevalier, N. Executive function development: making sense of the environment to behave adaptively. *Curr. Dir. Psychol. Sci.* **24**, 363–368 (2015).

[42] Goodkind, M. et al. Identification of a common neurobiological substrate for mental illness. *JAMA Psychiatry* **72**, 305–315 (2015). 25651064 10.1001/jamapsychiatry.2014.2206 PMC4791058

[43] Shanmugan, S. et al. Common and dissociable mechanisms of executive system dysfunction across psychiatric disorders in youth. *Am. J. Psychiatry* **173**, 517–526 (2016). 26806874 10.1176/appi.ajp.2015.15060725 PMC4886342

[44] Openneer, T. J. C. et al. Executive function in children with Tourette syndrome and attention-deficit/hyperactivity disorder: cross-disorder or unique impairments? *Cortex* **124**, 176–187 (2020). 31901563 10.1016/j.cortex.2019.11.007

[45] Adólfsdóttir, S., Sørensen, L. & Lundervold, A. J. The attention network test: a characteristic pattern of deficits in children with ADHD. *Behav. Brain Funct.* **4**, 9 (2008). 18269768 10.1186/1744-9081-4-9 PMC2265730

[46] Stuss, D. T. & Knight R. T. (eds) *Principles of Frontal Lobe Function* (Oxford Univ. Press, 2013)

[47] Kumar, U., Arya, A. & Agarwal, V. Neural network connectivity in ADHD children: an independent component and functional connectivity analysis of resting state fMRI data. *Brain Imaging Behav.* **15**, 157–165 (2021). 31903529 10.1007/s11682-019-00242-0

[48] Shallice, T. et al. Executive function profile of children with attention deficit hyperactivity disorder. *Dev. Neuropsychol.* **21**, 43–71 (2002). 12058835 10.1207/S15326942DN2101_3

[49] Petersen, S. E. & Posner, M. I. The attention system of the human brain: 20 years after. *Annu. Rev. Neurosci.* **35**, 73–89 (2012). 22524787 10.1146/annurev-neuro-062111-150525 PMC3413263

[50] Greene, C. M., Braet, W., Johnson, K. A. & Bellgrove, M. A. Imaging the genetics of executive function. *Biol. Psychol.* **79**, 30–42 (2008). 18178303 10.1016/j.biopsycho.2007.11.009

[51] Craig, F. et al. A review of executive function deficits in autism spectrum disorder and attention-deficit/hyperactivity disorder. *Neuropsychiatr. Dis. Treat.* **12**, 1191–1202 (2016). 27274255 10.2147/NDT.S104620 PMC4869784

[52] Yerys, B. E. et al. Neural correlates of set-shifting in children with autism. *Autism Res.* **8**, 386–397 (2015). 25599972 10.1002/aur.1454 PMC4508240

[53] Yerys, B. E. et al. Set-shifting in children with autism spectrum disorders: reversal shifting deficits on the Intradimensional/Extradimensional Shift Test correlate with repetitive behaviors. *Autism* **13**, 523–538 (2009). 19759065 10.1177/1362361309335716 PMC3018342

[54] Astle, D. E., Holmes, J., Kievit, R. & Gathercole, S. E. Annual Research Review: the transdiagnostic revolution in neurodevelopmental disorders. *J. Child Psychol. Psychiatry* **63**, 397–417 (2022). 34296774 10.1111/jcpp.13481

[55] Crisci, G., Caviola, S., Cardillo, R. & Mammarella, I. C. Executive functions in neurodevelopmental disorders: comorbidity overlaps between attention deficit and hyperactivity disorder and specific learning disorders. *Front. Hum. Neurosci.* **15**, 594234 (2021). 33732121 10.3389/fnhum.2021.594234 PMC7958764

[56] Willcutt, E. G., Doyle, A. E., Nigg, J. T., Faraone, S. V. & Pennington, B. F. Validity of the executive function theory of attention-deficit/hyperactivity disorder: a meta-analytic review. *Biol. Psychiatry* **57**, 1336–1346 (2005). 15950006 10.1016/j.biopsych.2005.02.006

[57] Liberati, A. et al. The PRISMA statement for reporting systematic reviews and meta-analyses of studies that evaluate healthcare interventions: explanation and elaboration. *Brit. Med. J.* **339**, b2700 (2009). 19622552 10.1136/bmj.b2700 PMC2714672

[58] Page, M. J. et al. The PRISMA 2020 statement: an updated guideline for reporting systematic reviews. *Brit. Med. J.* **372**, n71 (2021). 33782057 10.1136/bmj.n71 PMC8005924

[59] Howlin, P., Charman, T. & Ghaziuddin, M. *The SAGE Handbook of Developmental Disorders* (SAGE, 2011).

[60] Covidence systematic review software (Veritas Health Innovation, 2024).

[61] Landis, J. R. & Koch, G. G. The measurement of observer agreement for categorical data. *Biometrics* **33**, 159–174 (1977). 843571

