# Supplementary material for: Executive function in children with neurodevelopmental conditions: a systematic review and meta-analysis
Source: Nat Hum Behav. 2024 Oct 18;8(12):2357–66. doi: 10.1038/s41562-024-02000-9 (PMC11659155; doi:10.1038/s41562-024-02000-9)
Supplement: Supplementary file 1 — Supplementary Figs. 1 and 2 and Tables 1–8. [file 41562_2024_2000_MOESM1_ESM.pdf]

nature human behaviour

Supplementary information https://doi.org/10.1038/s41562-024-02000-9

Executive function in children with neurodevelopmental conditions: a systematic review and meta-analysis

In the format provided by the authors and unedited

Supplementary Table 1: Search Strategy

Medline_NDDs	1. (executive dysfunction or central executive).tw.<br>2. inhibitory Control.tw.<br>3. (Attention or updating).tw.<br>4. cognitive flexibility.tw.<br>5. mental flexibility.tw.<br>6. set switching.tw.<br>7. task switching.tw.<br>8. set shifting.tw.<br>9. short term memory.tw.<br>10. working memory.tw.<br>11. (fluency or Verbal Fluency).tw.<br>12. (planning and executive function).tw. [mp=title, book title, abstract, original title, name of substance word, subject heading word, floating sub-heading word, keyword heading word, organism supplementary concept word, protocol supplementary concept word, rare disease supplementary concept word, unique identifier, synonyms, population supplementary concept word, anatomy supplementary concept word]<br>13. cognitive planning.tw.<br>14. response inhibition.tw.<br>15. halstead reitan neuropsychological battery/ or luria nebraska neuropsychological battery/<br>16. (neuropsychological assessment and executive function).tw. [mp=title, book title, abstract, original title, name of substance word, subject heading word, floating sub-heading word, keyword heading word, organism supplementary concept word, protocol supplementary concept word, rare disease supplementary concept word, unique identifier, synonyms, population supplementary concept word, anatomy supplementary concept word]<br>17. behavior rating inventory of executive function.tw.<br>18. behavior rating inventory of executive function.tw.

19. BRIEF-P.tw.
20. Tower of london.tw.
21. (Stroop Color Word Test or stroop test).tw.
22. delis-kaplan executive function system.tw.
23. trail making test.tw.
24. luria-nebraska neuropsychological battery.tw.
25. task shifting.tw.
26. Go no-go task.tw.
27. (planning and executive function).tw.
28. Wisconsin Card Sorting Test.tw.
29. (delis-kaplan executive function system or DKEFS).tw.
30. Tower of london.tw.
31. Affective decision making.tw.
32. Childrens gambling task.tw.
33. Iowa gambling task.tw.
34. Sandbox task.tw.
35. (Cambridge Neuropsychological Test Automated Battery or CANTAB).tw.
36. Cambridge Gambling Task.tw.
37. Information Sampling Task.tw.
38. Tower of Hanoi.tw.
39. Hayling test.tw.
40. Eriksen flanker test.tw.
41. Color-Word interference test.tw.
42. NIH toolbox cognition battery.tw.
43. (Flanker Inhibitory Control and Attention test).tw.
44. List Sorting Working Memory Test.tw.
45. *Wechsler Memory Scale/ or wechsler memory scale.tw.
46. Digits backwards.tw.
47. n-back test.tw.
48. Letter Sequencing task.tw.
49. Intra-extra Dimensional Set-Shift.tw.
50. (Stop Signal Task and Stroop Stepping Test).tw.
51. Spatial Working Memory Test.tw.

52. Dimensional Change Card Sort Test.tw.
53. Flexible Item Selection Task.tw.
54. (Barkley Deficits in Executive Functioning Scale or BDEFS).tw.
55. (Controlled Oral Word Association Test or COWAT).tw.
56. (Behavioural Assessment of the Dysexecutive Syndrome or BADS).tw.
57. Dysexecutive Questionnaire.tw.
58. (Autism Spectrum disorder or ASD).tw.
59. autism spectrum disorders.tw.
60. Asperger Syndrome.tw.
61. Pervasive developmental Disorder.tw.
62. 58 or 59 or 60 or 61
63. tic disorder.tw.
64. tourette's syndrome.tw.
65. Tourettes disease.tw.
66. cerebral palsy.tw.
67. Down syndrome.tw.
68. Trisomy 21 syndrome.tw.
69. *Fragile X Syndrome/ or fragile X syndrome.tw.
70. Martin-Bell syndrome.tw.
71. williams syndrome.tw.
72. (williams beuren syndrome or Williams syndrome).tw.
73. *Prader Willi Syndrome/ or Prader Willi syndrome.tw.
74. Angelman syndrome.tw.
75. happy puppet syndrome.tw.
76. *Rett Syndrome/ or Rett syndrome.tw.
77. *Turners Syndrome/ or Turner syndrome.tw.
78. Smith-Magenis syndrome.tw.
79. *"Sclerosis (Nervous System)"/ or Tuberous sclerosis.tw.
80. DiGeorge syndrome.tw.
81. velocardiofacial syndrome.tw.
82. 22q11 deletion syndrome.tw.

83. dyslexia.tw. or *Dyslexia/
84. (dyscalculia or Acalculia).tw.
85. (Learning Disorders or Specific learning disorders).tw.
86. mental retardation.tw.
87. intellectual disability.tw.
88. intellectual developmental disorder.tw.
89. global developmental delay.tw.
90. reading disorder.tw.
91. mathematics disorder.tw.
92. disorder of written expression.tw.
93. motor skills disorder.tw.
94. developmental coordination disorder.tw.
95. stereotypic movement disorder.tw.
96. communication disorder.tw.
97. language disorder.tw.
98. phonological disorder.tw.
99. stuttering.tw.
100. childhood disintegrative disorder.tw.
101. speech sound disorder.tw.
102. childhood-onset fluency disorder.tw.
103. specific developmental disorder.tw.
104. (executive adj2 function*).tw. [mp=title, book title, abstract, original title, name of substance word, subject heading word, floating sub-heading word, keyword heading word, organism supplementary concept word, protocol supplementary concept word, rare disease supplementary concept word, unique identifier, synonyms, population supplementary concept word, anatomy supplementary concept word]
105. concept formation.tw.
106. (fluency or verbal fluency or non verbal fluency).tw.
107. "Delay of Gratification".tw.
108. (Attention and executive function).tw. [mp=title, book title, abstract, original title, name of substance word, subject heading word, floating sub-heading word, keyword heading

word, organism supplementary concept word, protocol supplementary concept word, rare disease supplementary concept word, unique identifier, synonyms, population supplementary concept word, anatomy supplementary concept word]

109. (Cognition and executive function).tw. [mp=title, book title, abstract, original title, name of substance word, subject heading word, floating sub-heading word, keyword heading word, organism supplementary concept word, protocol supplementary concept word, rare disease supplementary concept word, unique identifier, synonyms, population supplementary concept word, anatomy supplementary concept word]

110. working memory.tw.

111. Problem Solving.mp. and executive function.tw. [mp=title, book title, abstract, original title, name of substance word, subject heading word, floating sub-heading word, keyword heading word, organism supplementary concept word, protocol supplementary concept word, rare disease supplementary concept word, unique identifier, synonyms, population supplementary concept word, anatomy supplementary concept word]

112. Attention Deficit Hyperactivity Disorder.tw.

113. "attention deficit and disruptive behavior disorders"/ or attention deficit disorder with hyperactivity/ or ADHD.tw.

114. 112 or 113

115. Fetal Alcohol Spectrum Disorders/ or FASD.tw. or foetal alcohol spectrum disorder.tw.

116. task switching/

117. executive function/

118. set shifting.tw.

119. task switching.tw.

120. executive functioning measures/

121. 1 or 4 or 5 or 6 or 7 or 8 or 9 or 10 or 11 or 12 or 13 or 14 or 15 or 16 or 17 or 18 or 19 or 20 or 21 or 22 or 23 or 24 or

25 or 26 or 27 or 28 or 29 or 30 or 31 or 32 or 33 or 34 or 35 or 36 or 37 or 38 or 39 or 40 or 41 or 42 or 43 or 44 or 45 or 46 or 47 or 48 or 49 or 50 or 51 or 52 or 53 or 54 or 55 or 56 or 57 or 62 or 104 or 105 or 106 or 107 or 108 or 109 or 110 or 111 or 117 or 118 or 119 or 120

122. 62 and 121

123. 114 and 121

124. 63 or 64 or 65 or 66 or 67 or 68 or 69 or 70 or 71 or 72 or 73 or 74 or 75 or 76 or 77 or 78 or 79 or 80 or 81 or 82 or 83 or 84 or 85

125. 122 and 124

126. 123 and 124

127. 125 or 126

128. 121 and 62 and (63 or 64 or 65 or 66 or 67 or 68 or 69 or 70 or 71 or 72 or 73 or 74 or 75 or 76 or 77 or 78 or 79 or 80 or 81 or 82 or 83 or 84 or 85 or 86 or 87 or 88 or 89 or 90 or 91 or 92 or 93 or 94 or 95 or 96 or 97 or 98 or 99 or 100 or 101 or 102 or 103 or 114 or 115)

129. 121 and 63 and (62 or 64 or 65 or 66 or 67 or 68 or 69 or 70 or 71 or 72 or 73 or 74 or 75 or 76 or 77 or 78 or 79 or 80 or 81 or 82 or 83 or 84 or 85 or 86 or 87 or 88 or 89 or 90 or 91 or 92 or 93 or 94 or 95 or 96 or 97 or 98 or 99 or 100 or 101 or 102 or 103 or 114 or 115)

130. 121 and 64 and (62 or 63 or 65 or 66 or 67 or 68 or 69 or 70 or 71 or 72 or 73 or 74 or 75 or 76 or 77 or 78 or 79 or 80 or 81 or 82 or 83 or 84 or 85 or 86 or 87 or 88 or 89 or 90 or 91 or 92 or 93 or 94 or 95 or 96 or 97 or 98 or 99 or 100 or 101 or 102 or 103 or 114 or 115)

131. 121 and 65 and (62 or 63 or 64 or 66 or 67 or 68 or 69 or 70 or 71 or 72 or 73 or 74 or 75 or 76 or 77 or 78 or 79 or 80 or 81 or 82 or 83 or 84 or 85 or 86 or 87 or 88 or 89 or 90 or 91 or 92 or 93 or 94 or 95 or 96 or 97 or 98 or 99 or 100 or 101 or 102 or 103 or 114 or 115)

132. 121 and 66 and (62 or 63 or 64 or 65 or 67 or 68 or 69 or 70 or 71 or 72 or 73 or 74 or 75 or 76 or 77 or 78 or 79 or 80

or 81 or 82 or 83 or 84 or 85 or 86 or 87 or 88 or 89 or 90 or 91 or 92 or 93 or 94 or 95 or 96 or 97 or 98 or 99 or 100 or 101 or 102 or 103 or 114 or 115)

133. 121 and 67 and (62 or 63 or 64 or 65 or 66 or 68 or 69 or 70 or 71 or 72 or 73 or 74 or 75 or 76 or 77 or 78 or 79 or 80 or 81 or 82 or 83 or 84 or 85 or 86 or 87 or 88 or 89 or 90 or 91 or 92 or 93 or 94 or 95 or 96 or 97 or 98 or 99 or 100 or 101 or 102 or 103 or 114 or 115)

134. 121 and 68 and (62 or 63 or 64 or 65 or 66 or 67 or 69 or 70 or 71 or 72 or 73 or 74 or 75 or 76 or 77 or 78 or 79 or 80 or 81 or 82 or 83 or 84 or 85 or 86 or 87 or 88 or 89 or 90 or 91 or 92 or 93 or 94 or 95 or 96 or 97 or 98 or 99 or 100 or 101 or 102 or 103 or 114 or 115)

135. 121 and 69 and (62 or 63 or 64 or 65 or 66 or 67 or 68 or 70 or 71 or 72 or 73 or 74 or 75 or 76 or 77 or 78 or 79 or 80 or 81 or 82 or 83 or 84 or 85 or 86 or 87 or 88 or 89 or 90 or 91 or 92 or 93 or 94 or 95 or 96 or 97 or 98 or 99 or 100 or 101 or 102 or 103 or 114 or 115)

136. 121 and 70 and (62 or 63 or 64 or 65 or 66 or 67 or 68 or 69 or 71 or 72 or 73 or 74 or 75 or 76 or 77 or 78 or 79 or 80 or 81 or 82 or 83 or 84 or 85 or 86 or 87 or 88 or 89 or 90 or 91 or 92 or 93 or 94 or 95 or 96 or 97 or 98 or 99 or 100 or 101 or 102 or 103 or 114 or 115)

137. 121 and 71 and (62 or 63 or 64 or 65 or 66 or 67 or 68 or 69 or 70 or 72 or 73 or 74 or 75 or 76 or 77 or 78 or 79 or 80 or 81 or 82 or 83 or 84 or 85 or 86 or 87 or 88 or 89 or 90 or 91 or 92 or 93 or 94 or 95 or 96 or 97 or 98 or 99 or 100 or 101 or 102 or 103 or 114 or 115)

138. 121 and 72 and (62 or 63 or 64 or 65 or 66 or 67 or 68 or 69 or 70 or 71 or 73 or 74 or 75 or 76 or 77 or 78 or 79 or 80 or 81 or 82 or 83 or 84 or 85 or 86 or 87 or 88 or 89 or 90 or 91 or 92 or 93 or 94 or 95 or 96 or 97 or 98 or 99 or 100 or 101 or 102 or 103 or 114 or 115)

139. 121 and 73 and (62 or 63 or 64 or 65 or 66 or 67 or 68 or 69 or 70 or 71 or 72 or 74 or 75 or 76 or 77 or 78 or 79 or 80

or 81 or 82 or 83 or 84 or 85 or 86 or 87 or 88 or 89 or 90 or 91 or 92 or 93 or 94 or 95 or 96 or 97 or 98 or 99 or 100 or 101 or 102 or 103 or 114 or 115)

140. 121 and 74 and (62 or 63 or 64 or 65 or 66 or 67 or 68 or 69 or 70 or 71 or 72 or 73 or 75 or 76 or 77 or 78 or 79 or 80 or 81 or 82 or 83 or 84 or 85 or 86 or 87 or 88 or 89 or 90 or 91 or 92 or 93 or 94 or 95 or 96 or 97 or 98 or 99 or 100 or 101 or 102 or 103 or 114 or 115)

141. 121 and 75 and (62 or 63 or 64 or 65 or 66 or 67 or 68 or 69 or 70 or 71 or 72 or 73 or 74 or 76 or 77 or 78 or 79 or 80 or 81 or 82 or 83 or 84 or 85 or 86 or 87 or 88 or 89 or 90 or 91 or 92 or 93 or 94 or 95 or 96 or 97 or 98 or 99 or 100 or 101 or 102 or 103 or 114 or 115)

142. 121 and 76 and (62 or 63 or 64 or 65 or 66 or 67 or 68 or 69 or 70 or 71 or 72 or 73 or 74 or 75 or 77 or 78 or 79 or 80 or 81 or 82 or 83 or 84 or 85 or 86 or 87 or 88 or 89 or 90 or 91 or 92 or 93 or 94 or 95 or 96 or 97 or 98 or 99 or 100 or 101 or 102 or 103 or 114 or 115)

143. 121 and 77 and (62 or 63 or 64 or 65 or 66 or 67 or 68 or 69 or 70 or 71 or 72 or 73 or 74 or 75 or 76 or 78 or 79 or 80 or 81 or 82 or 83 or 84 or 85 or 86 or 87 or 88 or 89 or 90 or 91 or 92 or 93 or 94 or 95 or 96 or 97 or 98 or 99 or 100 or 101 or 102 or 103 or 114 or 115)

144. 121 and 78 and (62 or 63 or 64 or 65 or 66 or 67 or 68 or 69 or 70 or 71 or 72 or 73 or 74 or 75 or 76 or 77 or 79 or 80 or 81 or 82 or 83 or 84 or 85 or 86 or 87 or 88 or 89 or 90 or 91 or 92 or 93 or 94 or 95 or 96 or 97 or 98 or 99 or 100 or 101 or 102 or 103 or 114 or 115)

145. 121 and 79 and (62 or 63 or 64 or 65 or 66 or 67 or 68 or 69 or 70 or 71 or 72 or 73 or 74 or 75 or 76 or 77 or 78 or 80 or 81 or 82 or 83 or 84 or 85 or 86 or 87 or 88 or 89 or 90 or 91 or 92 or 93 or 94 or 95 or 96 or 97 or 98 or 99 or 100 or 101 or 102 or 103 or 114 or 115)

146. 121 and 80 and (62 or 63 or 64 or 65 or 66 or 67 or 68 or 69 or 70 or 71 or 72 or 73 or 74 or 75 or 76 or 77 or 78 or 79

or 81 or 82 or 83 or 84 or 85 or 86 or 87 or 88 or 89 or 90 or 91 or 92 or 93 or 94 or 95 or 96 or 97 or 98 or 99 or 100 or 101 or 102 or 103 or 114 or 115)

147. 121 and 81 and (62 or 63 or 64 or 65 or 66 or 67 or 68 or 69 or 70 or 71 or 72 or 73 or 74 or 75 or 76 or 77 or 78 or 79 or 80 or 82 or 83 or 84 or 85 or 86 or 87 or 88 or 89 or 90 or 91 or 92 or 93 or 94 or 95 or 96 or 97 or 98 or 99 or 100 or 101 or 102 or 103 or 114 or 115)

148. 121 and 82 and (62 or 63 or 64 or 65 or 66 or 67 or 68 or 69 or 70 or 71 or 72 or 73 or 74 or 75 or 76 or 77 or 78 or 79 or 80 or 81 or 83 or 84 or 85 or 86 or 87 or 88 or 89 or 90 or 91 or 92 or 93 or 94 or 95 or 96 or 97 or 98 or 99 or 100 or 101 or 102 or 103 or 114 or 115)

149. 121 and 83 and (62 or 63 or 64 or 65 or 66 or 67 or 68 or 69 or 70 or 71 or 72 or 73 or 74 or 75 or 76 or 77 or 78 or 79 or 80 or 81 or 82 or 84 or 85 or 86 or 87 or 88 or 89 or 90 or 91 or 92 or 93 or 94 or 95 or 96 or 97 or 98 or 99 or 100 or 101 or 102 or 103 or 114 or 115)

150. 121 and 84 and (62 or 63 or 64 or 65 or 66 or 67 or 68 or 69 or 70 or 71 or 72 or 73 or 74 or 75 or 76 or 77 or 78 or 79 or 80 or 81 or 82 or 83 or 85 or 86 or 87 or 88 or 89 or 90 or 91 or 92 or 93 or 94 or 95 or 96 or 97 or 98 or 99 or 100 or 101 or 102 or 103 or 114 or 115)

151. 121 and 85 and (62 or 63 or 64 or 65 or 66 or 67 or 68 or 69 or 70 or 71 or 72 or 73 or 74 or 75 or 76 or 77 or 78 or 79 or 80 or 81 or 82 or 83 or 84 or 86 or 87 or 88 or 89 or 90 or 91 or 92 or 93 or 94 or 95 or 96 or 97 or 98 or 99 or 100 or 101 or 102 or 103 or 114 or 115)

152. 121 and 86 and (62 or 63 or 64 or 65 or 66 or 67 or 68 or 69 or 70 or 71 or 72 or 73 or 74 or 75 or 76 or 77 or 78 or 79 or 80 or 81 or 82 or 83 or 84 or 85 or 87 or 88 or 89 or 90 or 91 or 92 or 93 or 94 or 95 or 96 or 97 or 98 or 99 or 100 or 101 or 102 or 103 or 114 or 115)

153. 121 and 87 and (62 or 63 or 64 or 65 or 66 or 67 or 68 or 69 or 70 or 71 or 72 or 73 or 74 or 75 or 76 or 77 or 78 or 79

or 80 or 81 or 82 or 83 or 84 or 85 or 86 or 88 or 89 or 90 or 91 or 92 or 93 or 94 or 95 or 96 or 97 or 98 or 99 or 100 or 101 or 102 or 103 or 114 or 115)

154. 121 and 88 and (62 or 63 or 64 or 65 or 66 or 67 or 68 or 69 or 70 or 71 or 72 or 73 or 74 or 75 or 76 or 77 or 78 or 79 or 80 or 81 or 82 or 83 or 84 or 85 or 86 or 87 or 89 or 90 or 91 or 92 or 93 or 94 or 95 or 96 or 97 or 98 or 99 or 100 or 101 or 102 or 103 or 114 or 115)

155. 121 and 89 and (62 or 63 or 64 or 65 or 66 or 67 or 68 or 69 or 70 or 71 or 72 or 73 or 74 or 75 or 76 or 77 or 78 or 79 or 80 or 81 or 82 or 83 or 84 or 85 or 86 or 87 or 88 or 90 or 91 or 92 or 93 or 94 or 95 or 96 or 97 or 98 or 99 or 100 or 101 or 102 or 103 or 114 or 115)

156. 121 and 90 and (62 or 63 or 64 or 65 or 66 or 67 or 68 or 69 or 70 or 71 or 72 or 73 or 74 or 75 or 76 or 77 or 78 or 79 or 80 or 81 or 82 or 83 or 84 or 85 or 86 or 87 or 88 or 89 or 91 or 92 or 93 or 94 or 95 or 96 or 97 or 98 or 99 or 100 or 101 or 102 or 103 or 114 or 115)

157. 121 and 91 and (62 or 63 or 64 or 65 or 66 or 67 or 68 or 69 or 70 or 71 or 72 or 73 or 74 or 75 or 76 or 77 or 78 or 79 or 80 or 81 or 82 or 83 or 84 or 85 or 86 or 87 or 88 or 89 or 90 or 92 or 93 or 94 or 95 or 96 or 97 or 98 or 99 or 100 or 101 or 102 or 103 or 114 or 115)

158. 121 and 92 and (62 or 63 or 64 or 65 or 66 or 67 or 68 or 69 or 70 or 71 or 72 or 73 or 74 or 75 or 76 or 77 or 78 or 79 or 80 or 81 or 82 or 83 or 84 or 85 or 86 or 87 or 88 or 89 or 90 or 91 or 93 or 94 or 95 or 96 or 97 or 98 or 99 or 100 or 101 or 102 or 103 or 114 or 115)

159. 121 and 93 and (62 or 63 or 64 or 65 or 66 or 67 or 68 or 69 or 70 or 71 or 72 or 73 or 74 or 75 or 76 or 77 or 78 or 79 or 80 or 81 or 82 or 83 or 84 or 85 or 86 or 87 or 88 or 89 or 90 or 91 or 92 or 94 or 95 or 96 or 97 or 98 or 99 or 100 or 101 or 102 or 103 or 114 or 115)

160. 121 and 94 and (62 or 63 or 64 or 65 or 66 or 67 or 68 or 69 or 70 or 71 or 72 or 73 or 74 or 75 or 76 or 77 or 78 or 79

or 80 or 81 or 82 or 83 or 84 or 85 or 86 or 87 or 88 or 89 or 90 or 91 or 92 or 93 or 95 or 96 or 97 or 98 or 99 or 100 or 101 or 102 or 103 or 114 or 115)

161. 121 and 95 and (62 or 63 or 64 or 65 or 66 or 67 or 68 or 69 or 70 or 71 or 72 or 73 or 74 or 75 or 76 or 77 or 78 or 79 or 80 or 81 or 82 or 83 or 84 or 85 or 86 or 87 or 88 or 89 or 90 or 91 or 92 or 93 or 94 or 96 or 97 or 98 or 99 or 100 or 101 or 102 or 103 or 114 or 115)

162. 121 and 96 and (62 or 63 or 64 or 65 or 66 or 67 or 68 or 69 or 70 or 71 or 72 or 73 or 74 or 75 or 76 or 77 or 78 or 79 or 80 or 81 or 82 or 83 or 84 or 85 or 86 or 87 or 88 or 89 or 90 or 91 or 92 or 93 or 94 or 95 or 97 or 98 or 99 or 100 or 101 or 102 or 103 or 114 or 115)

163. 121 and 97 and (62 or 63 or 64 or 65 or 66 or 67 or 68 or 69 or 70 or 71 or 72 or 73 or 74 or 75 or 76 or 77 or 78 or 79 or 80 or 81 or 82 or 83 or 84 or 85 or 86 or 87 or 88 or 89 or 90 or 91 or 92 or 93 or 94 or 95 or 96 or 98 or 99 or 100 or 101 or 102 or 103 or 114 or 115)

164. 121 and 98 and (62 or 63 or 64 or 65 or 66 or 67 or 68 or 69 or 70 or 71 or 72 or 73 or 74 or 75 or 76 or 77 or 78 or 79 or 80 or 81 or 82 or 83 or 84 or 85 or 86 or 87 or 88 or 89 or 90 or 91 or 92 or 93 or 94 or 95 or 96 or 97 or 99 or 100 or 101 or 102 or 103 or 114 or 115)

165. 121 and 99 and (62 or 63 or 64 or 65 or 66 or 67 or 68 or 69 or 70 or 71 or 72 or 73 or 74 or 75 or 76 or 77 or 78 or 79 or 80 or 81 or 82 or 83 or 84 or 85 or 86 or 87 or 88 or 89 or 90 or 91 or 92 or 93 or 94 or 95 or 96 or 97 or 98 or 100 or 101 or 102 or 103 or 114 or 115)

166. 121 and 100 and (62 or 63 or 64 or 65 or 66 or 67 or 68 or 69 or 70 or 71 or 72 or 73 or 74 or 75 or 76 or 77 or 78 or 79 or 80 or 81 or 82 or 83 or 84 or 85 or 86 or 87 or 88 or 89 or 90 or 91 or 92 or 93 or 94 or 95 or 96 or 97 or 98 or 99 or 101 or 102 or 103 or 114 or 115)

167. 121 and 101 and (62 or 63 or 64 or 65 or 66 or 67 or 68 or 69 or 70 or 71 or 72 or 73 or 74 or 75 or 76 or 77 or 78 or

79 or 80 or 81 or 82 or 83 or 84 or 85 or 86 or 87 or 88 or 89 or 90 or 91 or 92 or 93 or 94 or 95 or 96 or 97 or 98 or 99 or 100 or 102 or 103 or 114 or 115)

168. 121 and 102 and (62 or 63 or 64 or 65 or 66 or 67 or 68 or 69 or 70 or 71 or 72 or 73 or 74 or 75 or 76 or 77 or 78 or 79 or 80 or 81 or 82 or 83 or 84 or 85 or 86 or 87 or 88 or 89 or 90 or 91 or 92 or 93 or 94 or 95 or 96 or 97 or 98 or 99 or 100 or 101 or 103 or 114 or 115)

169. 121 and 103 and (62 or 63 or 64 or 65 or 66 or 67 or 68 or 69 or 70 or 71 or 72 or 73 or 74 or 75 or 76 or 77 or 78 or 79 or 80 or 81 or 82 or 83 or 84 or 85 or 86 or 87 or 88 or 89 or 90 or 91 or 92 or 93 or 94 or 95 or 96 or 97 or 98 or 99 or 100 or 101 or 102 or 114 or 115)

170. 121 and 114 and (62 or 63 or 64 or 65 or 66 or 67 or 68 or 69 or 70 or 71 or 72 or 73 or 74 or 75 or 76 or 77 or 78 or 79 or 80 or 81 or 82 or 83 or 84 or 85 or 86 or 87 or 88 or 89 or 90 or 91 or 92 or 93 or 94 or 95 or 96 or 97 or 98 or 99 or 100 or 101 or 102 or 103 or 115)

171. 121 and 115 and (62 or 63 or 64 or 65 or 66 or 67 or 68 or 69 or 70 or 71 or 72 or 73 or 74 or 75 or 76 or 77 or 78 or 79 or 80 or 81 or 82 or 83 or 84 or 85 or 86 or 87 or 88 or 89 or 90 or 91 or 92 or 93 or 94 or 95 or 96 or 97 or 98 or 99 or 100 or 101 or 102 or 103 or 114)

172. 128 or 129 or 130 or 131 or 132 or 133 or 134 or 135 or 136 or 137 or 138 or 139 or 140 or 141 or 142 or 143 or 144 or 145 or 146 or 147 or 148 or 149 or 150 or 151 or 152 or 153 or 154 or 155 or 156 or 157 or 158 or 159 or 160 or 161 or 162 or 163 or 164 or 165 or 166 or 167 or 168 or 169 or 170 or 171

173. limit 172 to yr="1980 - current"

174. limit 173 to (childhood <birth to 12 years> or adolescence <13 to 17 years>)

Supplementary Table 2: Key Executive Function Domains and Related Measures

EF domain	Background Information on EF Domains	Examples of Key Measures Used to Assess this Domain
**Global EF abilities**	• Overall Executive function encompasses a range of processing including but not limited to working memory, response inhibition and flexibility.	• Behavior Rating Inventory of Executive Function (BRIEF; ages 5 to 18 years)[25]<br>• Behavior Rating Inventory of Executive Function–Preschool Version (BRIEF-P; ages 2 to 5 years)[26]<br>• Childhood Executive Functioning Inventory (CHEXI;[62] Global measure of EF in children)
**Concept formation/Set-shifting**<br>*The capacity to shift between mental processes to form new concepts and identify the conceptual relationships shared by stimuli* [63]	• Emerges in early childhood and matures in adolescence, [64]<br>• Functional peak observed in mid adolescence (17 years) followed by decline (18-19 years)[65]<br>• Adult levels of set shifting observed in 8-10 year olds[66] but also in adolescence[67]	• WCST (Wisconsin Card Sorting Test)[68]<br>• The Children's Cooking Task (CCT)[69]<br>• Task Switch[70]<br>• Vienna Test System Trail Making Test-B (VTS TMT-B)<br>• Delis-Kaplan Executive Function System (D-KEFS) Number-Letter Switching[71]<br>• Delis-Kaplan Executive Function System (D-KEFS) Design Fluency Switching[71]<br>• d2 Selective Attention Test – % errors[72]<br>• d2 Selective Attention Test – Total Correct[72]
**Mental flexibility/Set-switching**<br>*The capacity to switch between mental processes (multiple tasks, operations, or mental sets) in response to changing demands*[73,74]	• Emerges in early childhood and matures in adolescence[64,75]	• Trail Making Task B/Trails-P[76]<br>• Wechsler Intelligence Scale for Children, Fourth Edition (WISC-IV) - Letter-Number Sequencing[77]<br>• Shift from Digit Span Forward to Digit Span Backward (WISC-IV)[77]<br>• Word Order Subtest (K-ABC)[78]<br>• Children's Category Test (CCT)[79]

**Fluency**<br>*The capacity to generate novel ideas (ideational fluency) and responses (phonemic and semantic fluency[80]. May be assessed by verbal and non-verbal tasks.*	• Emerges in early childhood and matures in early adolescence,[64,75]<br><br>• Greatest period of development in early to mid- childhood (5-8) with continued improvement into early adulthood[81]	• Category fluency<br>• Letter fluency<br>• Verbal fluency test<br>• Animals category/Animal Naming Test<br>• Controlled Oral Word Association Test (COWA) Phonemic Cue[82]<br>• Controlled Oral Word Association Test (COWA) Semantic Cue[82]<br>• Controlled Oral Word Association Test (COWA) FAS[82]<br>• California Verbal Learning Test-II (CVLT-II) Semantic Clustering[83]<br>• California Verbal Learning Test-II (CVLT-II) Long Delay Recall[83]<br>• Delis-Kaplan Executive Function System (D-KEFS) Verbal Category Fluency[71]<br>• Delis-Kaplan Executive Function System (D-KEFS) Category Switching Fluency[71]<br>• Delis-Kaplan Executive Function System (D-KEFS) Letter Fluency[71]
**Planning**<br>*The capacity to execute a sequence of actions so that a desired goal is achieve[84].*	• Emerges and significantly develops in early childhood, some research suggests brief regression of skills in adolescence, matures in early adulthood[64,75]<br><br>• Significant improvement in late adolescence (15-19) with optimal performance in early adulthood (20-29)[66]<br><br>• Greatest period of development in early to mid-childhood (5-8) with continued improvement into early adulthood[81]	• Rey-Osterieth Complex Figure[85]<br>• ToL (Tower of London)[86]<br>• ToH (Tower of Hanoi)[87]<br>• Clock Drawing Test[88]<br>• Block Design Subtest (WPPSI-IV, WISC-IV)[77,89]<br>• Symbol Search and Symbol Coding (WISC-IV, WPPSI-IV)[77,89]

**Response Inhibition**<br>*The capacity to inhibit a previously learned response[73].*	• Emerges in early childhood, matures in late childhood to early adolescence[75]<br><br>• Greatest period of development in early to mid-childhood (5-8) with continued improvement into early adolescence[81]<br><br>• Adult levels of response inhibition achieved in late childhood (age 11)[67]	• Stroop Color Word Interference Test_C-W[90]<br>• Stroop Color Word Interference [90]<br>• NEPSY-II_Inhibition B[91]<br>• NEPSY-II_Inhibtion C[91]<br>• 5 Digit Test[92]<br>• *Go/NoGo Test[93]*<br>• Delis-Kaplan Executive Function System (D-KEFS) Color-Word Interference – Condition 3 (CWIT 3)[71]<br>• Delis-Kaplan Executive Function System (D-KEFS) Color-Word Interference – Condition 4 (CWIT 4)[71]<br>• Delis-Kaplan Executive Function System (D-KEFS) Color-Word Interference[71]<br>• d2 – Accuracy[94]<br>• d2 – Deviation[94]<br>• d2 – Percentage of mistakes[94]<br>• Interference trials
**Working Memory (WM)**<br>*The capacity to store and manipulate information in temporary short term storage for complex cognitive manipulations[84].*	• Emerges in early childhood and matures in early adolescence.[75,95]<br><br>• Peak improvement in late adolescence (15-19) maintained in early adulthood.[66]	• NEPSY-II_Word List Interfernce[91]<br>• Digit Span (Backward, Sequencing), Arithmetic, Letter-Number Sequencing (WISC-IV, WPPSI-IV)[77,89]<br>• Working memory scale (K-ABC)[78]<br>• Delis-Kaplan Executive Function System (D-KEFS) Number Sequencing[71]<br>• Connors Continuous Performance Test Version 3 (CPT-3) – Commissions subscale[96]<br>• Degraded Continuous Performance Test (Degraded CPT) – Commissions[96]<br>• Degraded Continuous Performance Test (Degraded CPT) – Omissions[96]<br>• Visual-Spatial Working Memory (Visual-Spatial WM)[77,89,97]<br>• Wechsler Memory Scale-Revised (WMS-R, WMS-IV) Digit Span Backwards[97]<br>• Wechsler Memory Scale-Revised (WMS-R, WMS-IV) Spatial Span Backwards[97]

		• Auditory-Verbal Working Memory (Auditory-Verbal WM)[97]<br>• Digit Span Backwards Score[77]<br>• Letter Number Sequencing Test[77]<br>• Brief Visuospatial Memory Test-Revised (BVMT-R) Total Recall[98]<br>• Reading Span – Partial-Credit Unit (PCU) Score[99]<br>• Reading Span – Sentence Errors[99]
**Attention**<br>*The subjective experience of attending to environmental stimuli by people thought to have introspection and can demonstrate alertness.*[100]	• Emerges in newborns and is a mechanism that continues to develop into childhood.[101]<br><br>• Attention can be divided into two main forms: sustained and divided attention.<br><br>• Sustained attention refers to attentional focus performed over a sustained time-period.[100]<br><br>• Focused attention is where certain environmental stimuli is given a priority over others and the attentional processes serve task demands that engage certain cognitive resources.[100]	• TOVA[102]<br>• TEA-Ch[103]<br>• Test of Attentional Performance for Children (KiTAP)[104]<br>• Letter-Number Sequencing (WISC-IV)[77,89]<br>• Verbal span tasks (WISC-IV)[77,89]<br>• Word order subtest (K-ABC)[78]<br>• Test of Attentional Performance (TAP)[105]<br>• d2 test of attention[94]

Note: Seven key EF domains in table adapted with permission from Dr Eleni Demetriou[22]

Supplementary Table 3. Characteristics of Final Included Studies with a Neurodevelopmental Group versus Controls

Study Name & Year	Diagnostic Group	EF Domains Analysed	Hedges' *g* (95% CI)	Control Sample size	Age Range	Gender Distribution (% of Males)	JBI Quality Assessment
Albajara Saenz et al, 2020	ADHD (n=18) and ASD (n=13)	Response Inhibition	0.28 (-0.45, 1.00)	14	8-12 years	78.3	Good
Ayyildiz et al, 2021	ADHD (n=37) and ASD (n=33)	Global Domains of EF<br>Planning<br>Response Inhibition<br>Set-Shifting<br>Working Memory	1.88 (1.30, 2.47)	33	6-17 years	82.7	Good
Bayliss et al, 2000	ADHD (n=15) and LD (n=12)	Set-Shifting<br>Response Inhibition<br>Attention	0.17 (-0.56, 0.90)	15	8-12 years	70	Good
Benson et al, 2023	ASD (n=20) and FASD (n=27)	Response Inhibition<br>Planning<br>Set-Shifting<br>Global Domains of EF<br>Working Memory	4.24 (3.30, 5.19)	45	6-15 years	67.8	Good
Bental et al, 2007	ADHD (n=13), ADHD+RD (n=27) and RD (n=17)	Fluency<br>Planning<br>Response Inhibition<br>Working Memory	0.31 (-0.31, 0.93)	23	7-11 years	100	Good
Berenguer et al, 2018	ADHD (n=35), ASD+ADHD (n=22) and ASD (n=30)	Response Inhibition<br>Planning<br>Set-Shifting<br>Working Memory	1.73 (1.15, 2.31)	37	7-11 years	92.3	Good

Study Name & Year	Diagnostic Group	EF Domains Analysed	Hedges' *g* (95% CI)	Control Sample size	Age Range	Gender Distribution (% of Males)	JBI Quality Assessment
Brandimonte et al, 2011	ASD (n=10) and ADHD (n=10)	Response Inhibition	0.67 (-0.22, 1.56)	10	6-12 years	81.7	Good
Brankaer et al, 2017	22q11DS (n=25) and TS (n=24)	Working Memory	0.22 (-0.27, 0.70)	48	6-12 years	Not reported	Good
Carney et al, 2013	DS (n=25) and WS (n=24)	Working Memory<br>Fluency<br>Response Inhibition<br>Set-Shifting	0.18 (-0.37, 0.73)	26	8-18 years	42.9	Good
Carter Leno et al, 2018	ADHD (n=21) and ASD (n=37 to 41)	Response Inhibition<br>Set-Switching	0.45 (-0.04, 0.94)	42	10-16 years	73	Good
Caspersen et al, 2017	ADHD (n=24) and ASD (n=14)	Attention	0.44 (-0.10, 0.97)	57	8-12 years	76.8	Good
Chnstakou et al, 2013	ADHD (n=20) and ASD (n=20)	Attention	0.39 (-0.23, 1.01)	20	11-17 years	100	Good
Coles, 1997	FASD (n=15) and ADHD (n=17)	Set-Shifting<br>Attention	0.20 (-0.42, 0.82)	26	7-8 years	Not reported	Good
Corbett et al, 2009	ASD (n=18) and ADHD (n=18)	Set-Shifting<br>Fluency<br>Planning<br>Response Inhibition<br>Working Memory<br>Attention	0.53 (-0.14, 120)	18	7-12 years	Not reported	Good
Crippa, 2015	ADHD (n=11), ADHD+RD (n=13)	Set-Shifting<br>Planning<br>Response Inhibition<br>Attention	1.28 (0.63, 1.93)	71	7-12 years	75	Good

Study Name & Year	Diagnostic Group	EF Domains Analysed	Hedges' *g* (95% CI)	Control Sample size	Age Range	Gender Distribution (% of Males)	JBI Quality Assessment
Crisci et al, 2021	ADHD (n=18), ADHD+SLD (n=48) and SLD (n=18)	Response inhibition<br>Set-Shifting	0.87 (0.35, 1.38)	48	8-14 years	68	Good
Crisci & Mammeralla (Unpub)	ASD (n=50) and ADHD (n=64)	Attention	0.92 (0.57, 1.27)	94	8-16 years	86	Fair
Fernandez-Andres et al, 2019	ADHD (n=35), Dyslexia (n=35), ADHD+Dyslexia (n=35)	Set-Shifting<br>Planning<br>Response Inhibition<br>Working Memory	1.08 (0.57, 1.58)	35	8-10 years	48.6	Good
Fernandez-Andres et al, 2021	Dyslexia (n=35), ADHD (n=35), ADHD+Dyslexia (n=35)	Response Inhibition<br>Set-Shifting<br>Working Memory<br>Planning, Attention	1.21 (0.69, 1.72)	35	8-10 years	51.4	Good
Geurts, 2004	ASD (n=41) and ADHD (n=54)	Set-Shifting<br>Fluency<br>Planning<br>Response Inhibition<br>Working Memory<br>Attention	0.52 (0.10, 0.95)	41	6-13 years	Not reported	Good
Gioia et al, 2002	RD (n=34), ADHD-I (n=27), ADHD-C (n=26) and ASD (n=54)	Response Inhibition<br>Planning<br>Set-Shifting<br>Working Memory	1.50 (1.15, 1.85)	208	Not reported	72	Good
Glass et al, 2013	FASD (n=38) and ADHD (n=80)	Fluency<br>Planning<br>Set-Shifting<br>Response Inhibition<br>Working Memory	0.81 (0.49, 1.12)	136	8-16 years	136	Good

Study Name & Year	Diagnostic Group	EF Domains Analysed	Hedges' *g* (95% CI)	Control Sample size	Age Range	Gender Distribution (% of Males)	JBI Quality Assessment
Goldberg et al, 2005	ASD (n=17) and ADHD (n=21)	Set-Shifting<br>Planning<br>Response Inhibition<br>Working Memory	0.26 (-0.31, 0.82)	32	8-12 years	75	Good
Gooch et al, 2011	Dyslexia (n=17), ADHD+Dyslexia (n=24) and ADHD (n=17)	Response Inhibition<br>Attention	0.29 (-0.28, 0.83)	35	5-14 years	61	Good
Greimel et al, 2011	ADHD (n=23), TS (n=21) and ADHD+TS (n=25)	Set-Shifting<br>Response Inhibition<br>Attention	0.24 (-0.32, 0.79)	27	Up to 17 years	78.1	Good
Greimel et al, 2008	ADHD (n=20) and ADHD+TS (n=20)	Set-Shifting<br>Attention	0.35 (-0.27, 0.96)	20	8-15 years	50	Good
Hall et al, 1997	ADHD (n=14) and RD (n=17)	Attention	0.59 (-0.06, 1.24)	28	6-13 years	67	Good
Happé et al, 2006	ASD (n=32) and ADHD (n=30)	Set-Shifting<br>Fluency<br>Response Inhibition<br>Set-Shifting<br>Planning<br>Response Inhibition<br>Set-switching<br>Working Memory<br>Attention	0.42 (-0.08, 0.92)	32	8-16 years	100	Good
Hovik et al, 2016	TS (n=19) and ADHD (79)	Response Inhibition<br>Attention	0.17 (-0.28, 0.62)	50	8-17 years	60	Good
Hovik et al, 2015	ADHD (n=33) and TS (n=19)	Attention	0.42 (-0.06, 0.91)	50	Not reported	66	Good
Huang et al, 2016	ADHD (n=391) and ADHD+LD (n=380)	Set-Shifting<br>Response Inhibition	0.26 (0.09, 0.44)	188	6-14 years	Not reported	Good

Study Name & Year	Diagnostic Group	EF Domains Analysed	Hedges' *g* (95% CI)	Control Sample size	Age Range	Gender Distribution (% of Males)	JBI Quality Assessment
Hutchison et al, 2016	ADHD (n=21) and ASD (n=33)	Global domains of EF<br>Set-Shifting	1.42 (0.79, 2.06)	28	7-18 years	76.1	Good
Hwang-Gu, 2019	ASD (n=221), ASD+ADHD (n=97) and ADHD (n=8)	Attention	0.46 (0.24, 0.68)	249	8-15 years	60	Good
Ikeda et al, 2014	ID (n=11) and ASD+ID (n=9)	Response Inhibition	-0.36 (-1.10, 0.38)	21	7-18 years	50	Good
Jurgiel et al, 2023	ADHD (n=55), ADHD+PTD (n=33) and PTD (n=27)	Set-Shifting<br>Planning<br>Working Memory	1.60 (1.05, 2.16)	33	8-12 years	71.4	Good
Kado, 2020	ASD (n=69) and ADHD+ASD (n=43)	Set-Shifting	0.32 (-0.01, 0.66)	69	5-15 years	72	Good
Kado, 2012	PDD (n=52) and ADHD (n=46)	Set-Shifting	0.41 (0.02, 0.81)	52	5-15 years	78	Good
Karalunas et al, 2018	ASD (n=97) and ADHD (509)	Working Memory<br>Set-Switching<br>Response Inhibition	0.45 (0.26, 0.64)	301	Not reported	73.1	Good
Kibby et al, 2008	ADHD (n=30), ADHD+RD (n=30) and RD (n=23)	Working Memory	0.57 (0.04, 1.10)	30	6-15 years	Not reported	Good
Kirk, 2005	Turner syndrome (n=20) and Fragile X syndrome (12)	Working Memory	0.48 (-0.18, 1.13)	Turner syndrome control (n=90) and Fragile X syndrome control (n= 12)	7-12 years	Not reported	Good

Study Name & Year	Diagnostic Group	EF Domains Analysed	Hedges' *g* (95% CI)	Control Sample size	Age Range	Gender Distribution (% of Males)	JBI Quality Assessment
Kooistra, 2011	ADHD (n=47) and FASD (n=28)	Attention	0.08 (-0.40, 0.57)	38	7-10 years	51	Good
Kuhn et al, 2016	Dyscalculia (n=33) and ADHD (n=16)	Set-Shifting<br>Working Memory<br>Attention	0.51 (-0.01, 1.04)	40	Not reported	40	Good
Kuijper et al, 2017	ASD (n=36) and ADHD (n=34)	Working Memory<br>Response Inhibition	0.20 (-0.26, 0.67)	36	6-12 years	85.5	Fair
Kuijper et al, 2021	ASD (n=47) and ADHD (n=36)	Working Memory<br>Response Inhibition	0.23 (-0.21, 0.67)	38	6-12 years	87	Fair
Landerl et al, 2009	Dyslexia (n=21), Dyscalculia (n=20) and Dyslexia+Dyscalculia (n=26)	Working Memory	0.37 (-0.14, 0.89)	42	7-11 years	Not reported	Good
Lee, 2024	SLD (n=24), ADHD (n=30) and ADHD+RD (n=22)	Working Memory<br>Response Inhibition	1.58 (0.96, 2.20)	28	Not reported	Not reported	Good
Li et al, 2017	ASD (n=32) and ADHD (n= 58)	Working Memory	0.61 (0.17, 1.05)	39	6-16 years	100	Good
Lievore et al, 2024	ASD (n=60) and SLD (n=63)	Response Inhibition<br>Working Memory<br>Set-Shifting	0.04 (-0.26, 0.34)	140	8-16 years	Not reported	Good
Lievore & Mammarella (Unpub)	ASD (n=60) and Learning Disorders (n=80)	Set-Shifting	0.27 (-0.01, 0.56)	150	8-16 years	Not reported	Fair

**Study Name & Year**	**Diagnostic Group**	**EF Domains Analysed**	**Hedges' *g* (95% CI)**	**Control Sample size**	**Age Range**	**Gender Distribution (% of Males)**	**JBI Quality Assessment**
Lundervold et al, 2016	ASD (n=9), ASD+ADHD (n=11) and ADHD (n=38)	Attention	0.41 (-0.16, 0.98)	134	8-10 years	66	Fair
Maehler et al, 2016	Dyslexia (n=31), Dyslexia+ADHD (n=37), Dyscalculia (n=18), Dyscalculia+ADHD (n= 21), ADHD (n=34)	Set-Shifting<br>Fluency<br>Planning<br>Response Inhibition<br>Set-switching<br>Working Memory<br>Attention	0.47 (-0.05, 1.00)	31	Not reported	54	Good
Maghsoodloonejad et al, 2017	ADHD (n=36) and LD (n=47)	Response Inhibition<br>Attention	1.05 (0.58, 1.51)	43	7-12 years	Not reported	Fair
Mammarella, 2019	ASD (n=17) and LD (n=17)	Planning<br>Working Memory	0.77 (0.08, 1.47)	17	8-18 years	Not reported	Good
Martinussen et al, 2006	ADHD (n=60), LD (n=14) and ADHD+LD (n=28)	Set-Shifting<br>Fluency<br>Planning<br>Response Inhibition<br>Set-switching<br>Working Memory<br>Attention	0.98 (0.44, 1.53)	34	Not reported	58	Good
Marzocchi et al, 2008	ADHD (n= 35) and RD (n=22)	Set-Shifting<br>Fluency<br>Planning<br>Response Inhibition<br>Working Memory	0.44 (-0.08, 0.96)	30	7-12 years	86	Good
Matsuura et al, 2014	ASD (n=11) and ADHD (n=15)	Working Memory<br>Attention	0.31 (-0.40, 1.01)	19	Not reported	80	Good

Study Name & Year	Diagnostic Group	EF Domains Analysed	Hedges' *g* (95% CI)	Control Sample size	Age Range	Gender Distribution (% of Males)	JBI Quality Assessment
Maziero et al, 2020	Dyslexia (n=47), Developmental Coordination Disorder, DCD (n=22) and Dyslexia+DCD (n=27)	Working Memory	0.99 (0.48, 1.49)	42	7-12 years	Not reported	Good
Mettler, 2024	Dyslexia (n=81) and Dyslexia+DLD (n=43)	Working Memory	0.69 (0.38, 1.01)	165	7-9 years	53.6	Good
Mingozzi et al, 2024	SLD (n=61), MD (n=13) and RD+MD (n=14)	Working Memory<br>Response Inhibition	0.60 (0.09, 1.11)	90	Not reported	43.7	Good
Mohl, 2015	ADHD (n=14), ADHD+RD (n=10)	Attention	0.58 (-0.23, 1.40)	14	Not reported	100	Good
Moura et al, 2017	ADHD (n=32), Dyslexia (n=32), Dyslexia+ADHD (n=18)	Fluency<br>Planning<br>Set-switching<br>Working Memory	0.76 (0.23, 1.29)	34	8-10 years	72.8	Good
Mughal et al, 2020	ASD (n=21) and FASD (n=29)	Response Inhibition<br>Working Memory	0.54 (0.05, 1.04)	45	6-12 years	68.1	Good
Narhi, 1995	RD (n=21), ADHD+RD (n=25) and ADHD (n=17)	Set-switching	0.35 (-0.39, 1.09)	10	8-12 years	100	Good
Nyden et al, 1999	Asperger's (n=10), ADHD (n=10), RD/WD (n=10)	Set-Shifting<br>Response Inhibition<br>Working Memory	0.86 (-0.04, 1.75)	10	6-18 years	100	Good
Openneer et al, 2020	TS (n=34), TS+ADHD (n=26) and ADHD (n=54)	Set-Shifting<br>Response Inhibition<br>Working Memory	-0.08 (-0.49, 0.34)	60	8-12 years	76.5	Good

Study Name & Year	Diagnostic Group	EF Domains Analysed	Hedges' *g* (95% CI)	Control Sample size	Age Range	Gender Distribution (% of Males)	JBI Quality Assessment
Operto et al, 2021	ASD-HF (n = 19) ADHD (n = 21), SLD (n = 22)	Working Memory	0.96 (0.31, 1.61)	20	years	72.6	Good
Ozonoff et al, 1994	TS (n=14) and ASD (n=14)	Set-switching	0.12 (-0.60, 0.84)	14	8-16 years	83	Good
Ozonoff et al, 1999	ASD (n=40), TS (n=30) and ADHD (n=24)	Set-Shifting<br>Planning	0.33 (-0.18, 0.84)	29	8-17 years	Not reported	Good
Passolunghi et al, 2005	ADHD (n=10) and LD (n=10)	Working Memory	1.12 (0.21, 2.03)	10	9-11 years	Not reported	Good
Pennington et al, 1993	ADHD (n=16) and RD (n=15)	Set-Shifting<br>Planning<br>Attention	0.23 (-0.41, 0.88)	23	7-10 years	100	Good
Pereira et al, 2020	ADHD (n=11), RD (n=45) and ADHD/RD (n=15)	Fluency<br>Planning<br>Response Inhibition<br>Set-Switching<br>Working Memory	0.51 (-0.08, 1.11)	33	8-11 years	77.6	Good
Piek et al, 2007	DCD (n=18), ADHD-Inattentive (n=20) and ADHD (n=19)	Set-Switching<br>Set-Shifting	0.37 (-0.11, 0.86)	138	6-14 years	75.4	Good
Pitzianti et al, 2016	ASD (n=13), ASD+ADHD (n=12) and ADHD (n=13)	Planning<br>Response Inhibition<br>Working Memory	1.51 (0.62, 2.40)	13	8-15 years	92	Good
Poon et al, 2014	ADHD (n=27), RD (n=22) and ADHD+RD (n=34)	Planning<br>Response Inhibition<br>Working Memory	0.67 (0.11, 1.23)	25	12-18 years	100	Good
Poon et al, 2021	ADHD (n=29), SLD (n=78) and ADHD-I+RD (n=31)	Working Memory	0.71 (0.30, 1.13)	64	Not reported	62.3	Good

Study Name & Year	Diagnostic Group	EF Domains Analysed	Hedges' *g* (95% CI)	Control Sample size	Age Range	Gender Distribution (% of Males)	JBI Quality Assessment
Predescu, 2020	ADHD (n=27) and Borderline ID (n=16)	Attention<br>Planning<br>Information Processing	1.85 (1.20, 2.50)	42	6-11 years	Not reported	Fair
Pride, 2012	ADHD+NF1 (n=60) and NF1 (n=132)	Set-Shifting<br>Fluency<br>Planning, Attention	0.57 (0.22, 0.93)	52	6-16 years	54	Good
Purvis et al, 2000	ADHD (n=17), ADHD+RD (n=17) and RD (n=17)	Response Inhibition<br>Attention	0.63 (-0.05, 1.31)	17	7-11 years	74.5	Good
Quintero et al, 2014	22q11.2DS (n=32), Fragile X syndrome (n=24) and Turner syndrome (n=31)	Attention	1.18 (0.66, 1.71)	42	7-15 years	0	Good
Rhodes, 2011	ADHD (n=24) and WS (n=20)	Working Memory<br>Planning	1.64 (0.83, 2.46)	19	7-14 years	81.6	Fair
Roessner et al, 2007	TS (n=22), TD+ADHD (n=14) and ADHD (n=19)	Set-Shifting<br>Attention	0.33 (-0.29, 0.95)	22	Not reported	100	Good
Saito, 2019	ASD+ADHD (n=10) and ADHD (n=11)	Attention	0.95 (0.05, 1.86)	9	Not reported	90	Good
Salunkhe et al, 2021	ASD (n=21), ASD+ADHD (n=19) and ADHD (n=40)	Working Memory<br>Response Inhibition	0.29 (-0.22, 0.79)	40	Not reported	87.6	Good
Samyn et al, 2015	ADHD (n=30) and ASD (n=31)	Set-Shifting<br>Response Inhibition<br>Attention	0.14 (-0.25, 0.53)	148	10-15 years	Not reported	Good

Study Name & Year	Diagnostic Group	EF Domains Analysed	Hedges' *g* (95% CI)	Control Sample size	Age Range	Gender Distribution (% of Males)	JBI Quality Assessment
Samyn et al, 2014	ADHD (n=24) and ASD (n=20)	Response Inhibition	0.34 (-0.25, 0.94)	21	10-15 years	Not reported	Good
Samyn et al, 2017	ASD (n=25) and ADHD (n=25)	Attention	0.25 (-0.30, 0.80)	25	10-15 years	100	Good
Schuchardt, 2008	Dyscalculia (n=17), Dyscalculia+Dyslexia (n=20) and Dyslexia (n=30)	Working Memory	0.73 (0.15, 1.30)	30	7-10 years	61.1	Good
Schuchardt et al, 2013	Dyslexia (n=30), SLI+Dyslexia (n=16), CDSS (n=19) and SLI+CDSS (n=18)	Working Memory	1.05 (0.44, 1.65)	30	Not reported	60.2	Good
Schuerholz, 1996	ADHD+TS (n=19) and TS (n=21)	Fluency<br>Planning<br>Attention	0.29 (-0.29, 0.88)	27	6-14 years	86	Good
Schuerholz, 1998	ADHD (n=39), TS+ADHD (n=23) and TS (n=18)	Fluency<br>Attention	0.76 (0.23, 1.29)	36	6-16 years	52.7	Good
Seidman, 2001	ADHD (n=79), Dyslexia (n=16), Dyscalculia (n=32), Dyslexia+ Dyscalculia (n=21)	Set-Shifting, Planning, Response Inhibition, Working Memory, Attention	0.91 (-0.11, 0.48)	127	6-17 years	100	Good
Semrud-Clikeman et al, 2008	ADHD (n=39) and LD (n=16)	Planning<br>Attention	0.28 (-0.23, 0.80)	39	9-15 years	Not reported	Good

Study Name & Year	Diagnostic Group	EF Domains Analysed	Hedges' *g* (95% CI)	Control Sample size	Age Range	Gender Distribution (% of Males)	JBI Quality Assessment
Seidman, 2001	ADHD (n=79), Dyslexia (n=16), Dyscalculia (n=32), Dyslexia+ Dyscalculia (n=21)	Set-Shifting, Planning, Response Inhibition, Working Memory, Attention	0.91 (-0.11, 0.48)	127	6-17 years	100	Good
Semrud-Clikeman et al, 2010	ASD (n=50), ADHD (n=156) and NVLD (n=26)	Planning	0.59 (0.24, 0.94)	113	9-16 years	70	Good
Semrud-Clikeman et al, 2010a	ASD (n=15) and ADHD (n=49)	Set-Shifting<br>Planning<br>Response Inhibition<br>Set-switching<br>Working Memory	0.89 (0.33, 1.46)	32	9-16 years	55.4	Good
Semrud-Clikeman, 2014	ASD (n=37), NVLD (n=31)	Set-Shifting<br>Fluency<br>Planning<br>Set-switching<br>Working Memory	0.67 (0.20, 1.14)	40	8-17 years	82	Good
Shalev et al, 2019	Williams syndrome (n=25) and Downs Syndrome (n=18)	Attention	0.59 (0.11, 1.07)	99	3-7 years	Not reported	Good
Shanahan et al, 2006	ADHD (n=105), ADHD+RD (n=51) and RD (n=95)	Set-switching	0.69 (0.40, 0.98)	144	Not reported	57.9	Good
Shin et al, 2001	ADHD (n=21) and TS (n=16)	Set-switching<br>Attention	0.94 (0.27, 1.61)	22	6-18 years	Not reported	Good
Shin et al, 2003	ADHD (n=15), LD (n=13), ADHD+LD (n=15) and TS (n=15)	Planning	0.04 (-0.62, 0.71)	20	6-13 years	Not reported	Good

Study Name & Year	Diagnostic Group	EF Domains Analysed	Hedges' *g* (95% CI)	Control Sample size	Age Range	Gender Distribution (% of Males)	JBI Quality Assessment
Sinzig et al, 2008	ASD (n=20), ASD+ADHD (n=21) and ADHD (n=30)	Response Inhibition<br>Attention	0.26 (-0.28, 0.81)	30	6-18 years	90	Good
Sinzig et al, 2008a	ASD (n=20), ASD+ADHD (n=20) and ADHD (n=20)	Set-Shifting<br>Planning<br>Response Inhibition<br>Working Memory	0.23 (-0.39, 0.85)	20	6-18 years	88.4	Good
Sinzig 2014	ASD (n=26) and ADHD (n=30)	Response Inhibition<br>Attention<br>Set-Shifting	-0.01 (-0.53, 0.51)	29	4-9 years	85.4	Good
Slaby et al, 2023	DD (n=40) and ADHD+TD (n=22)	Global Domains of EF	0.73 (0.18, 1.27)	20	7-11 years	43.5	Good
Stanford et al, 2020	ADHD (n=20) and DLD (n=20)	Attention<br>Working Memory<br>Set-Switching	0.67 (0.03, 1.30)	20	6-12 years	62.5	Good
Stubenrauch, 2014	ADHD (n=21), ADHD+RD (n=17) and RD (n=22)	Response Inhibition	0.47 (-0.13, 1.07)	24	8-12 years	74.1	Good
Sukhodolsky et al, 2010	ADHD (n=64), TS (n=56) and ADHD+TS (n=45)	Attention<br>Response Inhibition	0.40 (0.05, 0.76)	71	Not reported	80.4	Good
Termine et al, 2016	ADHD (n=39), TS (n=13) and TS+ADHD (n=8)	Planning	0.92 (0.31, 1.52)	66	6-15 years	96.7	Good
Thornton et al, 2018	ADHD (n=20), DCD (n=9) and ADHD+DCD (n=18)	Response Inhibition	0.50 (-0.19, 1.19)	20	8-17 years	83	Good

Study Name & Year	Diagnostic Group	EF Domains Analysed	Hedges' *g* (95% CI)	Control Sample size	Age Range	Gender Distribution (% of Males)	JBI Quality Assessment
Tiffin-Richards, 2008	ADHD (n=20), ADHD+Dyslexia (n=20) and Dyslexia (n=20)	Set-Shifting Working Memory	0.90 (0.25, 1.55)	19	10-14 years	79	Good
Tsuchiya et al, 2005	ADHD (n=22) and ASD (n=17)	Set-Shifting	1.03 (0.41, 1.66)	25	Not reported	92.5	Good
Turker et al, 2019	ADHD (n=43), ADHD+RD (n=15) and RD (n=27)	Working Memory	0.75 (0.28, 1.21)	89	8-18 years	78.3	Good
Tye et al, 2014	ADHD (n=18), ASD+ADHD (n=29) and ASD (n=19)	Response Inhibition	0.26 (-0.33, 0.85)	26	13-18 years	100	Good
Unterrainer et al, 2016	ASD (n=18), ADHD (n=42) and ASD+ADHD (n=19-23)	Planning	0.05 (-0.46, 0.57)	42	6-14 years	100	Good
Van De Voorde et al, 2010	ADHD (n=19), RD (n=17), ADHD+RD (n=21)	Response Inhibition Working Memory	1.01 (0.29, 1.74)	19	8-12 years	72.7	Good
Van De Voorde et al, 2011	ADHD (n=19), RD (n=17), ADHD+RD (n=21)	Response Inhibition	0.55 (-0.10, 1.20)	19	8-12 years	72.7	Good
Wang et al, 2018	ADHD (n=30), RD (n=33), ADHD+RD (n=28)	Response Inhibition Working Memory	1.01 (0.47, 1.55)	30	Not reported	58.3	Good
Wang et al, 2021	ASD (n=24), ADHD (n=23) and ASD+ADHD (n=23)	Set-Shifting	1.07 (0.47, 1.67)	28	6-12 years	87.1	Good

Study Name & Year	Diagnostic Group	EF Domains Analysed	Hedges' *g* (95% CI)	Control Sample size	Age Range	Gender Distribution (% of Males)	JBI Quality Assessment
Ware, 2012	PAE (n=142) and ADHD (n=82)	Set-Shifting<br>Fluency,<br>Response Inhibition	0.85 (0.58, 1.12)	133	8-18 years	63.7	Good
Weyandt et al, 1994	ADHD (n=36) and DLD (n=34)	Working Memory.<br>Fluency, Set-Shifting,<br>Response Inhibition<br>Planning	0.28 (-0.16, 0.72)	45	6-12 years	77.1	Good
Willcutt, 2005	ADHD (n=113), ADHD+RD (n=64) and RD (n=109)	Set-Shifting<br>Response Inhibition<br>Set-Switching<br>Working Memory	0.62 (0.35, 0.88)	151	8-18 years	59.8	Good
Williams et al, 2013	ADHD (n=14), DCD (n=10) and ADHD+DCD (not reported)	Attention<br>Working Memory	0.64 (-0.10, 1.38)	18	7-12 years	70	Good
Winsler et al, 2007	ASD (n=33) and ADHD (not reported)	Set-Shifting<br>Set-Switching<br>Global Domains of EF	1.26 (0.68, 1.84)	28	7-18 years	79.4	Good
Woodcock, 2009	PWS (n=27 to 28) and Fragile X syndrome (n=20 to 28)	Attention	1.32 (0.71, 1.92)	28	5-19 years	71.4	Good
Xiao et al, 2012	ADHD (n=16) and ASD (n=19)	Response Inhibition	0.48 (-0.20, 1.16)	16	8-14 years	100	Good
Yang et al, 2009	ADHD (n=26) and ASD (n=20)	Set-Shifting<br>Response Inhibition<br>Working Memory	0.36 (-0.19, 0.90)	30	3-15 years	87.3	Good

Study Name & Year	Diagnostic Group	EF Domains Analysed	Hedges' *g* (95% CI)	Control Sample size	Age Range	Gender Distribution (% of Males)	JBI Quality Assessment
Yasumura et al, 2014	ASD (n=11) and ADHD (n=10)	Response Inhibition	0.45 (-0.33, 1.23)	15	Not reported	71.4	Good
Zarchi, 2014	Velocardiofacial (22q11.2 deletion) (n=39) and Williams (7q11.23 deletion) syndromes (n=24)	Set-Shifting<br>Response Inhibition<br>Working Memory<br>Response Inhibition<br>Set-Shifting	0.48 (-0.07, 1.02)	22	Not reported	47.9	Good

Supplementary Table 4. List of Excluded Studies at the Stage of Statistical Analysis and Reasons for Exclusion

Study Authors	Title	Exclusion reason
Hovik KT, Egeland J, Isquith PK, et al 2014	Distinct Patterns of Everyday Executive Function Problems Distinguish Children with Tourette Syndrome From Children With ADHD or Autism Spectrum Disorders.	Data screening deemed it to be an outlier, removed to ensure statistical integrity
Raldiris TL, Bowers TG, Towsey C. 2018	Comparisons of Intelligence and Behavior in Children With Fetal Alcohol Spectrum Disorder and ADHD	Data screening deemed it to be an outlier, removed to ensure statistical integrity
de Jong CGW, Van De Voorde S, Roeyers H, Raymaekers R, Oosterlaan J, Sergeant JA. 2009	How Distinctive Are ADHD And RD? Results Of a Double Dissociation Study.	Data screening deemed it to be an outlier, removed to ensure statistical integrity
Kibby MY, Newsham G, Imre Z, Schlak JE. 2021	Is Executive Dysfunction a Potential Contributor To The Comorbidity Between Basic Reading Disability And Attention-Deficit/Hyperactivity Disorder?	Data screening deemed it to be an outlier, removed to ensure statistical integrity
Holingue C, Volk H, Crocetti D, Gottlieb B, Spira AP, Mostofsky SH. 2021	Links between Parent-Reported Measures of Poor Sleep and Executive Function in Childhood Autism and Attention Deficit Hyperactivity Disorder	Data screening deemed it to be an outlier, removed to ensure statistical integrity

Supplementary Table 5 Overall effect for comorbidity NDC groups versus Controls

Comparison	Sample Size	Effect Size<br>Hedges' g (95% CIs)	*P*	τ^2
ADHD+SLD	(N= 28, k= 203)	0.80 (0.65,0.95)	<0.001	0.15
ASD +ADHD	(n=11, k=98)	0.63 (0.25, 1.02)	0.001	0.40
ADHD+TD	(n=9, k=58)	0.61 (0.19, 1.04)	0.004	0.39
**Less than three studies, analysis was not conducted**				
NF1 +ADHD (n=1), ASD +ID (n=1), CD +SLD (n=2), SLD+MD (n=2), FASD+ADHD (n=1), ADHD+MD (n=1), ADHD+PAE (n=1)				

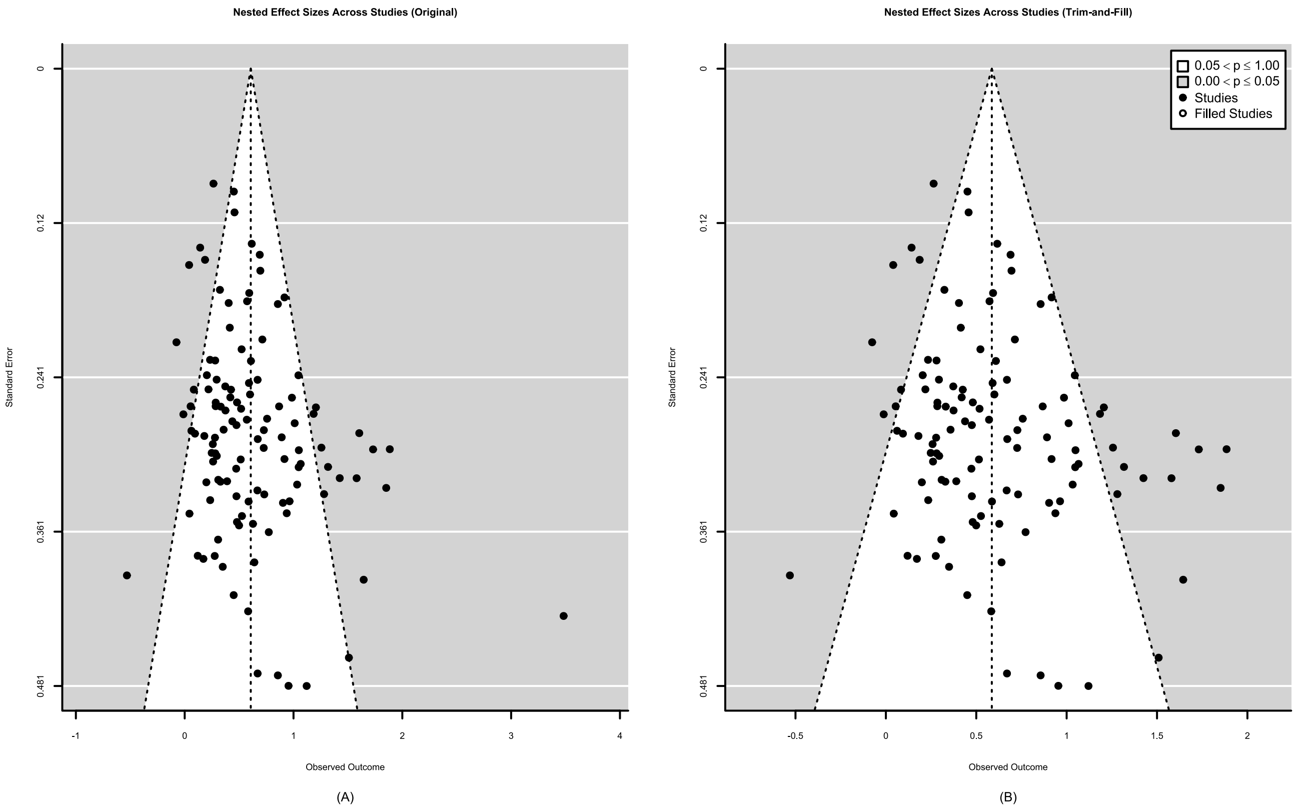

Supplementary Figure 1. Funnel Plot for Asymmetry Analysis with Trim and Fill Imputations. A. All eligible studies included. B. outlier (g>2) removed. The vertical dashed line represents the nested mean effect size in each reported study, with the outer dashed lines illustrating the 95% confidence intervals. Grey shaded areas indicate statistically significant differences (*P*<0.050), while the white area indicates non-significant differences (*P*>0.050). The *P*-values reported from the random-effects models are two-sided.

Supplementary Table 6. Characteristics of Final Included Studies Comparing Two Neurodevelopmental Groups

ADHD and DCD

Study Name & Year	Diagnostic Group	EF Domains Analysed	Hedges' *g* (95% CI)	Age Range	Gender Distribution (% of Males)	JBI Quality Assessment
Williams et al, 2013	ADHD (n=14) and DCD (n=10)	Attention	-0.25 (-1.04, 0.53)	7-12 years	70	Good
Thornton et al, 2018	ADHD (n=20) and DCD (n=9)	Response Inhibition	0.039 (-0.73, 0.81)	8-17 years	83	Good
Piek et al, 2007	DCD (n=18) and ADHD (n=19)	Set-Switching<br><br>Set-Shifting	-0.84 (-1.51, -0.17)	6-14 years	75.4	Good

ADHD and ASD

Study Name & Year	Diagnostic Group	EF Domains Analysed	Hedges' *g* (95% CI)	Age Range	Gender Distribution (% of Males)	JBI Quality Assessment
Gioia et al, 2002	ADHD (n=53) and ASD (n=54)	Response Inhibition<br>Planning<br>Set-Shifting<br>Working Memory	0.25 (-0.14, 0.64)	Not reported	72	Good
Semrud-Clikeman et al, 2010	ASD (n=50) and ADHD (n=156)	Planning	-0.46 (-0.78, -0.13)	9-16 years	70	Good
Happé et al, 2006	ASD (n=32) and ADHD (n=30)	Set-Shifting, Fluency, Response Inhibition, Set-Shifting, Planning, Response Inhibition, Set-switching, Working Memory, Attention	0.21 (-0.28, 0.71)	8-16 years	100	Good

Study Name & Year	Diagnostic Group	EF Domains Analysed	Hedges' *g* (95% CI)	Age Range	Gender Distribution (% of Males)	JBI Quality Assessment
Corbett et al, 2009	ASD (n=18) and ADHD (n=18)	Set-Shifting<br>Fluency<br>Planning<br>Response Inhibition<br>Working Memory<br>Attention	-0.65 (-1.33, 0.02)	7-12 years	Not reported	Good
Geurts, 2004	ASD (n=41) and ADHD (n=54)	Set-Shifting<br>Fluency<br>Planning<br>Response Inhibition<br>Working Memory<br>Attention	-0.31 (-0.72. 0.093)	6-13 years	Not reported	Good
Goldberg et al, 2005	ASD (n=17) and ADHD (n=21)	Set-Shifting<br>Planning<br>Response Inhibition<br>Working Memory	-0.24 (-0.87, 0.39)	8-12 years	75	Good
Matsuura et al, 2014	ASD (n=11) and ADHD (n=15)	Working Memory<br>Attention	-0.025 (-0.78, 0.73)	Not reported	80	Good
Ozonoff et al, 1999	ASD (n=40) and ADHD (n=24)	Set-Shifting<br>Planning	-0.43 (-0.94, 0.080)	8-17 years	Not reported	Good
Sinzig et al, 2008	ASD (n=20) and ADHD (n=30)	Response Inhibition<br>Attention	0.33 (-0.24, 0.89)	6-18 years	90	Good
Sinzig et al, 2008a	ASD (n=20) and ADHD (n=20)	Set-Shifting<br>Planning<br>Response Inhibition<br>Working Memory	0.22 (-0.40, 0.84)	6-18 years	88.4	Good
Sinzig 2014	ASD (n=26) and ADHD (n=30)	Response Inhibition<br>Attention,Set-Shifting	-0.28 (-0.80, 0.25)	4-9 years	85.4	Good

Study Name & Year	Diagnostic Group	EF Domains Analysed	Hedges' *g (*95% CI)	Age Range	Gender Distribution (% of Males)	JBI Quality Assessment
Tsuchiya et al, 2005	ADHD (n=22) and ASD (n=17)	Set-Shifting	0.050 (-0.58, 0.68)	Not reported	92.5	Good
Yang et al, 2009	ADHD (n=26) and ASD (n=20)	Set-Shifting<br>Response Inhibition<br>Working Memory	-0.26 (-0.84, 0.32)	3-15 years	87.3	Good
Xiao et al, 2012	ADHD (n=16) and ASD (n=19)	Response Inhibition	-0.15 (-0.80, 0.51)	8-14 years	100	Good
Tye et al, 2014	ADHD (n=18) and ASD (n=19)	Response Inhibition	0.20 (-0.46, 0.85)	13-18 years	100	Good
Samyn et al, 2015	ADHD (n=30) and ASD (n=31)	Set-Shifting<br>Response Inhibition<br>Attention	0.14 (-0.36, 0.64)	10-15 years	Not reported	Good
Samyn et al, 2014	ADHD (n=24) and ASD (n=20)	Response Inhibition	0.37 (-0.22, 0.96)	10-15 years	Not reported	Good
Semrud-Clikeman et al, 2010a	ASD (n=15) and ADHD (n=49)	Set-Shifting<br>Planning<br>Response Inhibition<br>Set-switching<br>Working Memory	-0.28 (-0.85, 0.3)	9-16 years	55.4	Good
Hwang-Gu, 2019	ASD (n=221) and ADHD (n=8)	Attention	0.29 (0.051, 0.53)	8-15 years	60	Good
Lundervold et al, 2016	ASD (n=9) and ADHD (n=38)	Attention	0.57 (-0.16, 1.30)	8-10 years	66	Fair

Study Name & Year	Diagnostic Group	EF Domains Analysed	Hedges' *g (*95% CI)	Age Range	Gender Distribution (% of Males)	JBI Quality Assessment
Pitzianti et al, 2016	ASD (n=13) and ADHD (n=13)	Planning<br>Response Inhibition<br>Working Memory	0.16 (-0.60, 0.92)	8-15 years	92	Good
Operto et al, 2021	ASD (n = 19) and ADHD (n = 21)	Working Memory	0.67 (0.048, 1.30)	years	72.6	Good
Brandimonte et al, 2011	ASD (n=10) and ADHD (n=10)	Response Inhibition	0.48 (-0.44, 1.40)	6-12 years	81.7	Good
Unterrainer et al, 2016	ASD (n=18) and ADHD (n=42)	Planning	0.50 (-0.088, 1.08)	6-14 years	100	Good
Crisci & Mammeralla (Unpub)	ASD (n=50) and ADHD (n=64)	Attention	-0.10 (-0.47, 0.27)	8-16 years	86	Fair
Albajara Saenz et al, 2020	ADHD (n=18) and ASD (n=13)	Response Inhibition	0.26 (-0.44, 0.96)	8-12 years	78.3	Good
Karalunas et al, 2018	ASD (n=97) and ADHD (509)	Working Memory<br>Set-Switching<br>Response Inhibition	0.0079 (-0.21, 0.22)	Not reported	73.1	Good
Wang et al, 2021	ASD (n=24) and ADHD (n=23)	Set-Shifting	-0.15 (-0.78, 0.48)	6-12 years	87.1	Good
Samyn et al, 2017	ASD (n=25) and ADHD (n=25)	Attention	0.19 (-0.37, 0.74)	10-15 years	100	Good
Salunkhe et al, 2021	ASD (n=21) and ADHD (n=40)	Working Memory<br>Response Inhibition	0.078 (-0.45, 0.60)	Not reported	87.6	Good

Study Name & Year	Diagnostic Group	EF Domains Analysed	Hedges' *g* (95% CI)	Age Range	Gender Distribution (% of Males)	JBI Quality Assessment
Kim & Song 2020	ADHD (n=44) and ASD (n=49)	Working Memory<br>Information Processing	0.067 (-0.34, 0.47)	6-15 years	75.7	Good
Ayyildiz et al, 2021	ADHD (n=37) and ASD (n=33)	Global Domains of EF, Planning, Response Inhibition, Set-Shifting, Working Memory	0.12 (-0.35, 0.58)	6-17 years	82.7	Good
Berenguer et al, 2018	ADHD (n=35) and ASD (n=30)	Response Inhibition<br>Planning<br>Set-Shifting<br>Working Memory	1.13 (0.60, 1.65)	7-11 years	92.3	Good
Kuijper et al, 2017	ASD (n=36) and ADHD (n=34)	Working Memory<br>Response Inhibition	-0.075 (-0.54, 0.39)	6-12 years	85.5	Fair
Kuijper et al, 2021	ASD (n=47) and ADHD (n=36)	Working Memory<br>Response Inhibition	0.040 (-0.39, 0.47)	6-12 years	87	Fair
Carter Leno et al, 2018	ADHD (n=21) and ASD (n=41)	Response Inhibition<br>Set-Switching	-0.14 (-0.67, 0.39)	10-16 years	73	Good
Caspersen et al, 2017	ADHD (n=24) and ASD (n=14)	Attention	0.36 (-0.29,1.02)	8-12 years	76.8	Good
Chnstakou et al, 2013	ADHD (n=20) and ASD (n=20)	Attention	0.18 (-0.43,0.79)	11-17 years	100	Good
Li et al, 2017	ASD (n=32) and ADHD (n= 58)	Working Memory	0.17 (-0.25, 0.60)	6-16 years	100	Good

Study Name & Year	Diagnostic Group	EF Domains Analysed	Hedges' *g* (95% CI)	Age Range	Gender Distribution (% of Males)	JBI Quality Assessment
Lundervold et al, 2016	ASD (n=9) and ADHD (n=38)	Attention	0.12 (-0.61, 0.85)	8-10 years	66	Fair
Hutchison et al, 2016	ADHD (n=21) and ASD (n=33)	Global domains of EF Set-Shifting	-0.13 (-0.67, 0.41)	7-18 years	76.1	Good
Yasumura et al, 2014	ASD (n=11) and ADHD (n=10)	Response Inhibition	0.48 (-0.37, 1.32)	Not reported	71.4	Good

ADHD and CTD

Study Name & Year	Diagnostic Group	EF Domains Analysed	Hedges' *g* (95% CI)	Age Range	Gender Distribution (% of Males)	JBI Quality Assessment
Greimel et al, 2011	ADHD (n=23) and CTD (n=21)	Attention<br>Response Inhibition<br>Set-Shifting	0.27 (-0.31. 0.86)	Up to 17 years	78.1	Good
Hovik et al, 2016	CTD (n=19) and ADHD (n=79)	Response Inhibition	0.19 (-0.31,0.68)	8-17 years	60	Good
Roessner et al, 2007	CTD (n=22) and ADHD (n=19)	Attention<br>Set-Shifting	0.57 (-0.047, 1.18)	Not reported	100	Good
Shin et al, 2001	ADHD (n=21) and CTD (n=16)	Attention<br>Set-Switching	1.25 (0.54, 1.95)	6-18 years	Not reported	Good
Shin et al, 2003	ADHD (n=15) and CTD (n=15)	Planning	0.42 (-0.29, 1.14)	6-13 years	Not reported	Good
Openneer et al, 2020	CTD (n=34) and ADHD (n=54)	Response Inhibition<br>Set-Shifting<br>Working Memory	0.27 (-0.16, 0.70)	8-12 years	76.5	Good

Study Name & Year	Diagnostic Group	EF Domains Analysed	Hedges' *g* (95% CI)	Age Range	Gender Distribution (% of Males)	JBI Quality Assessment
Ozonoff et al, 1999	CTD (n=30) and ADHD (n=24)	Set-Shifting<br>Planning	0.068 (-0.46, 0.60)	8-17 years	Not reported	Good
Hovik et al, 2015	ADHD (n=33) and CTD (n=19)	Attention	0.047 (-0.51,0.60)	Not reported	66	Good
Schuerholz, 1998	ADHD (n=39) and CTD (n=18)	Fluency<br>Attention	0.92 (0.33, 1.50)	6-16 years	52.7	Good
Sukhodolsky, 2010	ADHD (n=64) and CTD (n=56)	Attention<br>Response Inhibition	0.25 (-0.11, 0.60)	Not reported	80.4	Good
Termine et al, 2016	ADHD (n=39) and CTD (n=13)	Planning	0.28 (-0.35, 0.91)	6-15 years	96.7	Good
Jurgiel et al, 2023	ADHD (n=55) and CTD (n=27)	Set-Shifting<br>Planning<br>Working Memory	1.96 (1.40, 2.51)	8-12 years	71.4	Good

ADHD and SLD

Study Name & Year	Diagnostic Group	EF Domains Analysed	Hedges' *g (*95% CI)	Age Range	Gender Distribution (% of Males)	JBI Quality Assessment
Fernandez-Andres et al, 2019	ADHD (n=35) and SLD (n=35)	Set-Shifting<br>Planning<br>Response Inhibition<br>Working Memory	1.33 (0.81, 1.84)	8-10 years	48.6	Good
Gioia et al, 2002	SLD (n=34) and ADHD (n=53)	Response Inhibition<br>Planning<br>Set-Shifting<br>Working Memory	0.85 (0.40, 1.30)	Not reported	72	Good

Study Name & Year	Diagnostic Group	EF Domains Analysed	Hedges' *g* (95% CI)	Age Range	Gender Distribution (% of Males)	JBI Quality Assessment
Kuhn et al, 2016	SLD (n=33) and ADHD (n=16)	Set-Shifting<br>Working Memory<br>Attention	-0.38 (-0.98, 0.21)	Not reported	40	Good
Maehler et al, 2016	SLD (n=49) and ADHD (n=34)	Set-Shifting<br>Fluency<br>Planning<br>Response Inhibition<br>Set-switching<br>Working Memory<br>Attention	-0.02 (-0.45, 0.41)	Not reported	54	Good
Martinussen et al, 2006	ADHD (n=60) and SLD (n=14)	Set-Shifting<br>Fluency<br>Planning<br>Response Inhibition<br>Set-switching<br>Working Memory<br>Attention	-0.59 (-1.17, -0.01)	Not reported	58	Good
Marzocchi et al, 2008	ADHD (n= 35) and SLD (n=22)	Set-Shifting<br>Fluency<br>Planning<br>Response Inhibition<br>Working Memory	0.17 (-0.37, 0.70)	7-12 years	86	Good
Moura et al, 2017	ADHD (n=32) and SLD (n=32)	Fluency<br>Planning<br>Set-switching<br>Working Memory	0.03 (-0.46,0.52)	8-10 years	72.8	Good

Study Name & Year	Diagnostic Group	EF Domains Analysed	Hedges' *g* (95% CI)	Age Range	Gender Distribution (% of Males)	JBI Quality Assessment
Pereira et al, 2020	ADHD (n=11) and SLD (n=45)	Fluency<br>Planning<br>Response Inhibition<br>Set-Switching<br>Working Memory	-0.52 (-1.18, 0.15)	8-11 years	77.6	Good
Poon et al, 2014	ADHD (n=27) and SLD (n=22)	Planning<br>Response Inhibition<br>Working Memory	0.16 (-0.40, 0.72)	12-18 years	100	Good
Semrud-Clikeman et al, 2008	ADHD (n=39) and SLD (n=16)	Planning<br>Attention	0.24 (-0.33, 0.82)	9-15 years	Not reported	Good
Semrud-Clikeman et al, 2010	ADHD (n=156) and SLD (n=26)	Planning	1.04 (0.62,1.47)	9-16 years	70	Good
Shin et al, 2003	ADHD (n=15) and SLD (n=13)	Planning	0.18 (-0.56, 0.91)	6-13 years	Not reported	Good
Stubenrauch, 2014	ADHD (n=21) and SLD (n=22)	Response Inhibition	-0.14 (-0.73, 0.45)	8-12 years	74.1	Good
Willcutt, 2005	ADHD (n=113) and SLD (n=109)	Set-Shifting<br>Response Inhibition<br>Set-Switching<br>Working Memory	-0.13 (-0.39, 0.13)	8-18 years	59.8	Good
Gooch et al, 2011	SLD (n=17) and ADHD (n=17)	Response Inhibition<br>Attention	0.16 (-0.51, 0.83)	5-14 years	61	Good
Tiffin-Richards, 2008	ADHD (n=20) and SLD (n=20)	Set-Shifting<br>Working Memory	0 (-0.61, 0.61)	10-14 years	79	Good

Study Name & Year	Diagnostic Group	EF Domains Analysed	Hedges' *g* (95% CI)	Age Range	Gender Distribution (% of Males)	JBI Quality Assessment
Van De Voorde et al, 2010	ADHD (n=19) and SLD (n=17)	Response Inhibition<br>Working Memory	0.28 (-0.37, 0.92)	8-12 years	72.7	Good
Van De Voorde et al, 2011	ADHD (n=19) and SLD (n=17)	Response Inhibition	0.15 (-0.49, 0.80)	8-12 years	72.7	Good
Wang et al, 2018	ADHD (n=30) and SLD (n=33)	Response Inhibition<br>Working Memory	0.06 (-0.45, 0.56)	Not reported	58.3	Good
Narhi, 1995	SLD (n=21) and ADHD (n=17)	Set-switching	-0.19 (-0.81, 0.44)	8-12 years	100	Good
Shanahan et al, 2006	ADHD (n=105) and SLD (n=95)	Set-switching	0.26 (-0.02, 0.54)	Not reported	57.9	Good
Bayliss et al, 2000	ADHD (n=15) and SLD (n=12)	Set-Shifting<br>Response Inhibition<br>Attention	0.37 (-0.40,1.13)	8-12 years	70	Good
Bental et al, 2007	ADHD (n=13) and SLD (n=17)	Fluency<br>Planning<br>Response Inhibition<br>Working Memory	-0.09 (-0.80,0.62)	7-11 years	100	Good
Hall et al, 1997	ADHD (n=14) and SLD (n=17)	Attention	0.03 (-0.67, 0.72)	6-13 years	67	Good
Passolunghi et al, 2005	ADHD (n=10) and SLD (n=10)	Working Memory	0 (-0.84, 0.84)	9-11 years	Not reported	Good
Pennington et al, 1993	ADHD (n=16) and SLD (n=15)	Set-Shifting<br>Planning<br>Attention	0.70 (-0.01, 1.41)	7-10 years	100	Good

Study Name & Year	Diagnostic Group	EF Domains Analysed	Hedges' *g (*95% CI)	Age Range	Gender Distribution (% of Males)	JBI Quality Assessme nt
Purvis et al, 2000	ADHD (n=17) and SLD (n=17)	Response Inhibition<br>Attention	0.29 (-0.38, 0.95)	7-11 years	74.5	Good
Kibby et al, 2008	ADHD (n=30) and SLD (n=23)	Working Memory	-0.33 (-0.88, 0.22)	6-15 years	Not reported	Good
Maghsoodloonejad et al, 2017	ADHD (n=36) and SLD (n=47)	Response Inhibition<br>Attention	-0.79 (-1.25, -0.33)	7-12 years	Not reported	Fair
Turker et al, 2019	ADHD (n=43) and SLD (n=27)	Working Memory	-0.30 (-0.78, 0.18)	8-18 years	78.3	Good
Crisci et al, 2021	ADHD (n=18) and SLD (n=18)	Response inhibition<br>Set-Shifting	0.28 (-0.37, 0.92)	8-14 years	68	Good
Fernandez-Andres et al, 2021	SLD (n=35) and ADHD (n=35)	Response Inhibition<br>Set-Shifting<br>Working Memory<br>Planning<br>Attention	1.35 (0.84, 1.87)	8-10 years	51.4	Good
Operto et al, 2021	ADHD (n = 21) and SLD (n = 22)	Working Memory	-0.50 (-1.09, 0.10)	years	72.6	Good
Lee, 2024	SLD (n=24) and ADHD (n=30)	Working Memory<br>Response Inhibition	0.05 (-0.52, 0.62)	Not reported	Not reported	Good
Poon et al, 2021	ADHD (n=29) and SLD (n=78)	Working Memory	0.05 (-0.38, 0.47)	Not reported	62.3	Good

ASD and SLD

Study Name & Year	Diagnostic Group	EF Domains Analysed	Hedges' *g* (95% CI)	Age Range	Gender Distribution (% of Males)	JBI Quality Assessment
Gioia et al, 2002	SLD (n=34) and ASD (n=54)	Response Inhibition<br>Planning<br>Set-Shifting<br>Working Memory	0.59 (0.15, 1.03)	Not reported	72	Good
Semrud-Clikeman et al, 2010	ASD (n=50) and SLD (n=26)	Planning	0.62 (0.14, 1.09)	9-16 years	70	Good
Operto et al, 2021	ASD (n = 19) and SLD (n = 22)	Working Memory	-1.14 (-1.79, -0.49)	years	72.6	Good
Lievore et al, 2024	ASD (n=60) and SLD (n=63)	Response Inhibition<br>Working Memory<br>Set-Shifting	0.015 (-0.34, 0.37)	8-16 years	Not reported	Good

DS and WS

Study Name & Year	Diagnostic Group	EF Domains Analysed	Hedges' *g* (95% CI)	Age Range	Gender Distribution (% of Males)	JBI Quality Assessment
Shalev et al, 2019	Williams syndrome (n=25) and Downs Syndrome (n=18)	Attention	-0.25 (-1.11, 0.61)	3-7 years	Not reported	Good
Vicari et al, 2006	Williams syndrome (n=15) and Downs Syndrome (n=18)	Information Processing<br>Working Memory	0.54 (0.13, 0.96)	Not reported	63.65	Fair

**Study Name & Year**	**Diagnostic Group**	**EF Domains Analysed**	**Hedges' *g* (95% CI)**	**Age Range**	**Gender Distribution (% of Males)**	**JBI Quality Assessment**
Breckenridge et al 2013	Williams syndrome (n=32) and Downs Syndrome (n=32)	Working Memory<br>Attention<br>Sustained Attention	-0.29 (-0.56, -0.018)	Not reported	Not reported	Fair
Carney et al, 2013	DS (n=25) and WS (n=24)	Working Memory, Fluency, Response Inhibition, Set-Shifting	-0.16 (-0.48. 0.16)	8-18 years	42.9	Good

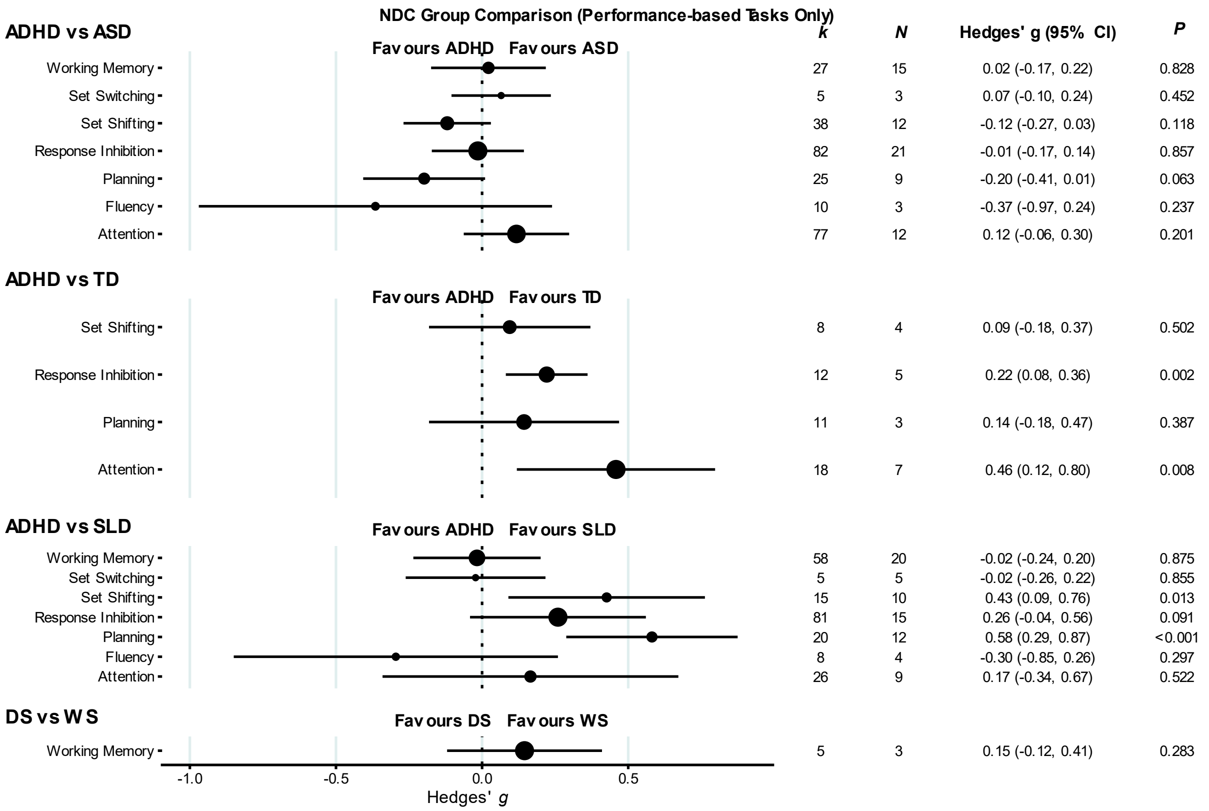
NDC Group Comparison (Performance-based Tasks Only)
ADHD vs ASD
k
N
Hedges' g (95% CI)
P
Favours ADHD
Favours ASD
Working Memory
27
15
0.02 (-0.17, 0.22)
0.828
Set Switching
5
3
0.07 (-0.10, 0.24)
0.452
Set Shifting
38
12
-0.12 (-0.27, 0.03)
0.118
Response Inhibition
82
21
-0.01 (-0.17, 0.14)
0.857
Planning
25
9
-0.20 (-0.41, 0.01)
0.063
Fluency
10
3
-0.37 (-0.97, 0.24)
0.237
Attention
77
12
0.12 (-0.06, 0.30)
0.201
ADHD vs TD
Favours ADHD
Favours TD
Set Shifting
8
4
0.09 (-0.18, 0.37)
0.502
Response Inhibition
12
5
0.22 (0.08, 0.36)
0.002
Planning
11
3
0.14 (-0.18, 0.47)
0.387
Attention
18
7
0.46 (0.12, 0.80)
0.008
ADHD vs SLD
Favours ADHD
Favours SLD
Working Memory
58
20
-0.02 (-0.24, 0.20)
0.875
Set Switching
5
5
-0.02 (-0.26, 0.22)
0.855
Set Shifting
15
10
0.43 (0.09, 0.76)
0.013
Response Inhibition
81
15
0.26 (-0.04, 0.56)
0.091
Planning
20
12
0.58 (0.29, 0.87)
<0.001
Fluency
8
4
-0.30 (-0.85, 0.26)
0.297
Attention
26
9
0.17 (-0.34, 0.67)
0.522
DS vs WS
Favours DS
Favours WS
Working Memory
5
3
0.15 (-0.12, 0.41)
0.283
-1.0
-0.5
0.0
0.5
Hedges' g

(A)

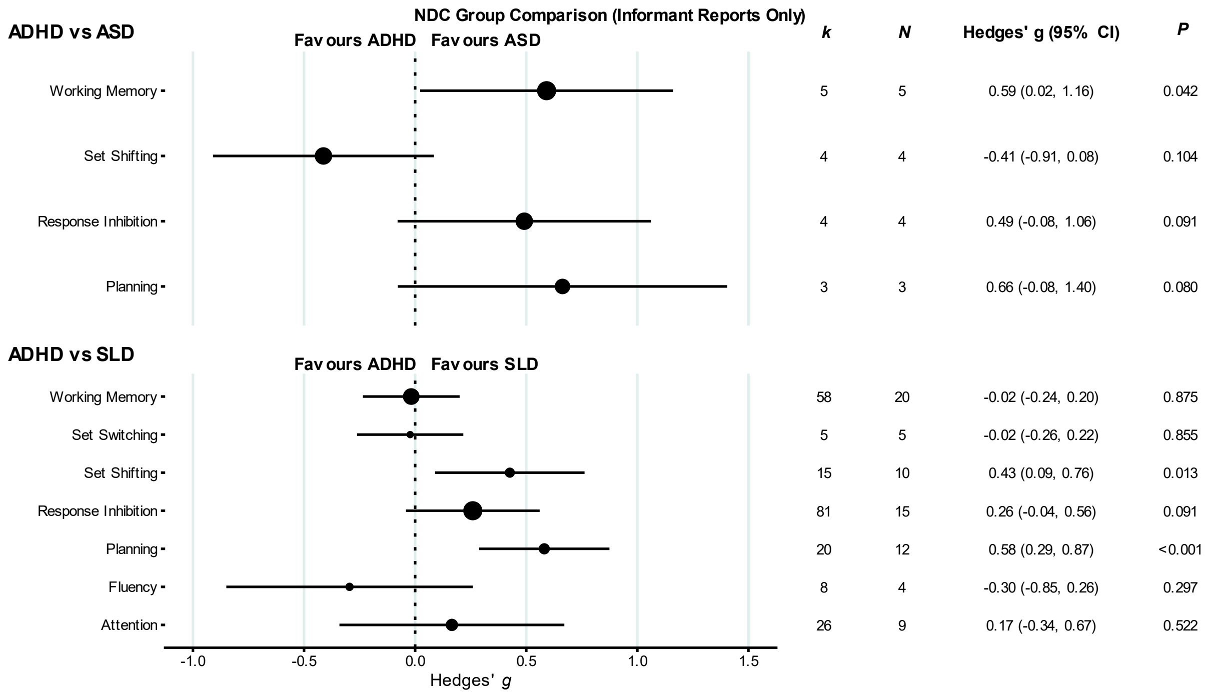

(B)

Supplementary Figure 2. Effect sizes for seven areas of EF across neurodevelopmental comparisons with (A) performance only, and (B) informant only measures. **Mean effects and their 95% confidence intervals are presented by the central black circle and the horizontal line. The size of the black circle reflects the number of studies included in each comparison. The dashed line at g= 0 indicates no effect, while *P*-values show whether the effect was statistically significant. The *P*-values reported from the random-effects models are two-sided.**

Supplementary Table 7. NDC conditions distribution across DSM editions.

Edition	Condition (%, number of study)
DSM-5<br><br>(N=47)	ADHD (34.04%, N=16), ASD (12.77%, N =6), SLD (19.1%, N =9), TD (2.13%, N =1), DD (2.13%, N =1), MD (2.13%, N =1), ASD+ADHD (6.38%, N =3), ADHD+TD (2.13%, N =1), ADHD+SLD (17.02%, N=8), SLD+MD (2.13%, N =1)
DSM-IV<br><br>(N= 168)	ADHD (38.69%, N=65), ASD (22.62%, N=38), SLD (11.90%, N=20), TD (4.17%, N=7), MD (1.79%, N=3), PAE (1.19%, N=2), FASD (1.79%, N=3), NF1 (0.60%, N=1), WS (0.60%, N=1), ASD+ADHD (4.17%, N =7), ADHD+TD (2.38%, N=4), ADHD+SLD (7.74%, N=13), ADHD+MD (0.60%, N=1), FASD+ADHD (0.60%, N=1), ADHD+PAE (0.60%, N=1), NF1+ADHD (0.60%, N=1)
DSM-III-R<br><br>(N=20)	ADHD (30%, N=6), ASD (5%, N=1), SLD (15%, N=3), TD (20%, N=4), PAE (5%, N=1), ADHD+TD (15%, N=3), ADHD+SLD (10%, N=2)

Supplementary Table 8. Number of studies comparing two NDC groups.

	ADHD	ASD	22q11DS	TD	FXS	WS	DS	FASD	CD	ID	SLD	PAE	DLD	MD	CP	PWS
**ADHD**	-	45	-	12	-	1	-	2	2	1	35	3	2	4	-	-
**ASD**	45	-	-	3	-	-	-	2	2	1	7	-	1	1	-	-s
**22q11DS**	-	-	-	2	1	-	-	-	-	-	-	-	-	-	-	-
**TD**	12	3	2	-	1	-	-	-	-	1	1	-	-	-	-	-
**FXS**	-	-	1	1	-	-	-	-	-	-	-	-	-	-	-	1
**WS**	1	-	-	-	-	-	3	-	-	-	-	-	-	-	-	1
**DS**	-	-	-	-	-	3	-	-	-	-	-	-	-	-	-	-
**FASD**	2	2	-	-	-	-	-	-	-	-	-	-	-	-	-	-
**CD**	2	2	-	-	-	-	-	-	-	-	-	-	-	-	-	-
**ID**	1	1	-	1	-	-	-	-	-	-	-	-	-	-	-	-
**SLD**	35	7	-	1	-	-	-	-	-	-	-	-	-	3	1	-
**PAE**	3	-	-	-	-	-	-	-	-	-	-	-	-	-	-	-
**DLD**	2	1	-	-	-	-	-	-	-	-	-	-	-	-	-	-
**MD**	4	1	-	-	-	-	-	-	-	-	3	-	-	-	-	-
**CP**	-	-	-	-	-	-	-	-	-	-	1	-	-	-	-	-
**PWS**	-	-	-	-	1	1	-	-	-	-	-	-	-	-	-	-

Note: CD: communication disorders, ID: intellectual disabilities, DLD: Developmental language delays, CP: cerebral palsy

62 Catale, C., Meulemans, T. & Thorell, L. B. The childhood executive function inventory: confirmatory factor analyses and cross-cultural clinical validity in a sample of 8- to 11-year-old children. *J Atten Disord* **19**, 489-495 (2015).

63 Delis, D. C., Kaplan, E. & Kramer, J. H. *D-KEFS Executive function system: Examiners Manual*. (Psychological Corporation, 2001).

64 Anderson, P. Assessment and development of executive function (EF) during childhood. *Child Neuropsychology* **8**, 71-82 (2002).

65 Taylor, S. J., Barker, L. A., Heavey, L. & McHale, S. The typical development trajectory of social and executive functions in late adolescence and early childhood. *Developmental Psychology* **49**, 1253-1265 (2013).

66 De Luca, C. R. *et al.* Normative data from the Cantab. I: development of executive function over the lifespan. *Journal of Clinical and Experimental Neuropsychology* **25**, 242-254 (2003).

67 Huizinga, M., Dolan, C. V. & van der Molen, M. W. Age related change in executive function: Developmental trends and a laten variable analysis. *Neuropsychologia* **44**, 2017-2036 (2006).

68 Grant, D. A. & Berg, E. A. Wisconsin card sorting test. *Journal of Experimental Psychology* (1993).

69 Fogel, Y. The Children's Cooking Task, a Performance-Based Assessment to Evaluate Executive Functions in Adolescents With Neurodevelopmental Disorders. *The American Journal of Occupational Therapy* **72**, 7211500019p7211500011-7211500019p7211500011 (2018).

70 Monsell, S. Task switching. *Trends in cognitive sciences* **7**, 134-140 (2003).

71 Delis, D. C., Kaplan, E. & Kramer, J. H. Delis-Kaplan executive function system. *The Psychological Corporation* (2001).

72 Bates, M. E. & Lemay, E. P. The d2 Test of attention: construct validity and extensions in scoring techniques. *Journal of the International Neuropsychological Society* **10**, 392-400 (2004).

73 Miyaki, A. *et al.* The unity and diversity of executive functions and their contributions to complex "frontal lobe" tasks: a latent variable analysis. *Cognitive Psychology* **41**, 49-100 (2000).

74 Pennington, B. F. & Ozonoff, S. Executive functions and developmental psychopathology. *Child Psychology & Psychiatry & Allied Disciplines* **37**, 51-87 (1996).

75 Hunter, S. J., Edidin, J. P. & Hinkle, C. D. in *Executive Function and Dysfunction* (eds S.J. Hunter & E.P. Sparrow) 17-36 (Cambridge Univ. Press, 2012).

76 Espy, K. A. & Cwik, M. F. The development of a trial making test in young children: the TRAILS-P. *Clin Neuropsychol* **18**, 411-422 (2004).

77 Wechsler, D. *Wechsler Intelligence Scale for Children–Fourth Edition.*, (The Psychological Corporation, 2003).

78 Lichtenberger, E. O. & Kaufman, A. S. in *Encyclopedia of Cross-Cultural School Psychology* (ed Caroline S. Clauss-Ehlers) 557-560 (Springer US, 2010).

79 Boll, T. J. *Children's category test: Slides for young children*. (Psychological Corporation, 1993).

80 Bishop, D. V. & Norbury, C. F. Executive functions in children with communication impairments, in relation to autistic symptomatology I: Generativity. *Autism* **9**, 7-27 (2005).

81 Romine, C. & Reynolds, C. A model of the development of frontal lobe functioning: findings from a meta-analysis. *Applied Neuropsychology* **12** (2005).

82 Patterson, J. in *Encyclopedia of Clinical Neuropsychology* (eds Jeffrey S. Kreutzer, John DeLuca, & Bruce Caplan) 703-706 (Springer New York, 2011).

83 Delis, D. C., Kramer, J. H., Kaplan, E., & Ober, B. A. . California Verbal Learning Test--Second Edition (CVLT –II) *APA PsycTests* (2000).

84 Lezak, M. D., Howieson, D. B., Bigler, E. D. & D., T. *Neuropsychological Assessment*. (2012).

85 Osterrieth, P. A. Le test de copie d'une figure complexe; contribution a l'etude de la perception et de la memoire. *Archives de psychologie* (1944).

86 Anderson, P., Anderson, V. & Lajoie, G. The tower of London test: Validation and standardization for pediatric populatons. *The Clinical Neuropsychologist* **10**, 54-65 (1996).

87 Welsh, M. C. & Huizinga, M. The development and preliminary validation of the Tower of Hanoi-Revised. *Assessment* **8**, 167-176 (2001).

88 Agrell, B. & Dehlin, O. The clock-drawing test. *Age and ageing* **27**, 399-404 (1998).

89 Wechsler, D. Wechsler preschool and primary scale of intelligence—fourth edition. *The Psychological Corporation San Antonio, TX* (2012).

90 Jensen, A. R. & Rohwer, W. D. The stroop color-word test: A review. *Acta Psychologica* **25**, 36-93 (1966).

91 Korkman, M. NEPSY: A proposed neuropsychological test battery for young developmentally-disabled children--theory and evaluation (1989).

92 Sedó, M. '5 digit test': a multilinguistic non-reading alternative to the Stroop test. *Revista de neurologia* **38**, 824-828 (2004).

93 Iverson, G. L. in *Encyclopedia of Clinical Neuropsychology* (eds Jeffrey S. Kreutzer, John DeLuca, & Bruce Caplan) 1162-1163 (Springer New York, 2011).

94 Brickenkamp, R., & Zilmer, E. 2 Test of Attention (d2) *APA PsycTests.* (1998).

95 Blakemore, S.-J. & Choudhury, S. Development of the adolescent brain: implications for executive function and social cognition. *Journal of Child Psychology and Psychiatry* **47**, 296-312 (2006).

96 Folsom, R. & Levin, P. in *Encyclopedia of Autism Spectrum Disorders* (ed Fred R. Volkmar) 783-787 (Springer New York, 2013).

97 Dzikon, C. in *The Wiley Encyclopedia of Personality and Individual Differences* 529-532 (2020).

98 Benedict, R. H. *Brief visuospatial memory test--revised*. (PAR, 1997).

99 Farmer, T. A., Fine, A. B., Misyak, J. B. & Christiansen, M. H. Reading Span Task Performance, Linguistic Experience, and the Processing of Unexpected Syntactic Events. *Quarterly Journal of Experimental Psychology* **70**, 413-433 (2017).

100 Cohen, R. A. *The neuropsychology of attention, 2nd ed*. (Springer Science + Business Media, 2014).

101 de Haan, M. & Johnson, M. Mechanisms and theories of brain development. *The cognitive neuroscience of development*, 1-18 (2003).

102 Greenberg, L. M. & Waldmant, I. D. Developmental Normative Data on The Test of Variables of Attention (T.O.V.A.™). *Journal of Child Psychology and Psychiatry* **34**, 1019-1030,(1993).

103 Evans, A. S. & Preston, A. S. in *Encyclopedia of Clinical Neuropsychology* (eds Jeffrey S. Kreutzer, John DeLuca, & Bruce Caplan) 2493-2493 (Springer New York, 2011).

104 Zimmermann P, G. M., Fimm B. . KiTAP Test of Attentional Performance for Children. *Psychologische Testsysteme* (2002).

105 Zimmermann, P. & Fimm, B. Test of attentional performance [German Version 2.2 (TAP)]. *Herzogenrath: Psytest;* (2009).
