# Supplementary material for: Executive function in children with neurodevelopmental conditions: a systematic review and meta-analysis
Source: Nat Hum Behav. 2024 Oct 18;8(12):2357–66. doi: 10.1038/s41562-024-02000-9 (PMC11659155; doi:10.1038/s41562-024-02000-9)
Supplement: Supplementary file 2 — Reporting Summary [file 41562_2024_2000_MOESM2_ESM.pdf]

nature portfolio

Corresponding author(s): Adam J. Guastella

Last updated by author(s): Aug 14, 2024

Reporting Summary

Nature Portfolio wishes to improve the reproducibility of the work that we publish. This form provides structure for consistency and transparency in reporting. For further information on Nature Portfolio policies, see our Editorial Policies and the Editorial Policy Checklist.

Statistics

For all statistical analyses, confirm that the following items are present in the figure legend, table legend, main text, or Methods section.

n/a	Confirmed	
☐	☒	The exact sample size (*n*) for each experimental group/condition, given as a discrete number and unit of measurement
☐	☒	A statement on whether measurements were taken from distinct samples or whether the same sample was measured repeatedly
☐	☒	The statistical test(s) used AND whether they are one- or two-sided<br>*Only common tests should be described solely by name; describe more complex techniques in the Methods section.*
☐	☒	A description of all covariates tested
☐	☒	A description of any assumptions or corrections, such as tests of normality and adjustment for multiple comparisons
☐	☒	A full description of the statistical parameters including central tendency (e.g. means) or other basic estimates (e.g. regression coefficient) AND variation (e.g. standard deviation) or associated estimates of uncertainty (e.g. confidence intervals)
☐	☒	For null hypothesis testing, the test statistic (e.g. *F*, *t*, *r*) with confidence intervals, effect sizes, degrees of freedom and *P* value noted<br>*Give P values as exact values whenever suitable.*
☒	☐	For Bayesian analysis, information on the choice of priors and Markov chain Monte Carlo settings
☐	☒	For hierarchical and complex designs, identification of the appropriate level for tests and full reporting of outcomes
☐	☒	Estimates of effect sizes (e.g. Cohen's *d*, Pearson's *r*), indicating how they were calculated

Our web collection on statistics for biologists contains articles on many of the points above.

Software and code

Policy information about availability of computer code

Data collection	Manuscript screening and data extraction were completed through Covidence (Extraction 1) (https://www.covidence.org) and a customised excel spreadsheet (Version 2404).
Data analysis	Meta-analysis was conducted through open source software, RStudio (2023.06.1 Build 524) and R (version 4.3.3), and customised codes are available on GitHub (https://github.com/CarterSunUSYD/Transdiagnostic_EF_meta.git).The package used to conduct the meta-analysis was metafor (Version 3.8-1), meta (Version 6.5-0), dplyr(Version 1.1.2), readxl (Version 1.4.3).

For manuscripts utilizing custom algorithms or software that are central to the research but not yet described in published literature, software must be made available to editors and reviewers. We strongly encourage code deposition in a community repository (e.g. GitHub). See the Nature Portfolio guidelines for submitting code & software for further information.

Data

Policy information about availability of data

All manuscripts must include a data availability statement. This statement should provide the following information, where applicable:

- Accession codes, unique identifiers, or web links for publicly available datasets
- A description of any restrictions on data availability
- For clinical datasets or third party data, please ensure that the statement adheres to our policy

The data used to undertake this systematic review and meta-analysis are freely available on GitHub (https://github.com/CarterSunUSYD/Transdiagnostic_EF_meta.git). Databases used in this study including MEDLINE, Embase and PsycINFO.

Research involving human participants, their data, or biological material

Policy information about studies with human participants or human data. See also policy information about sex, gender (identity/presentation), and sexual orientation and race, ethnicity and racism.

Reporting on sex and gender	The meta-analysis extracted gender information from the original text of each eligible studies.
Reporting on race, ethnicity, or other socially relevant groupings	This review did not explicitly extract race and ethnicity information for the quantitative meta-analysis.
Population characteristics	This review included all studies that measured executive functioning using established tests in participants under 18 years of age and describing populations comparing two or more paediatric neurodevelopmental conditions.
Recruitment	Participant demographics and diagnostic conditions were extracted directly from each included study. Manuscripts reporting on the same cohort were nested into one study group to reduce potential bias from repeating samples in the meta-analysis.
Ethics oversight	Not applicable for meta-analysis.

Note that full information on the approval of the study protocol must also be provided in the manuscript.

Field-specific reporting

Please select the one below that is the best fit for your research. If you are not sure, read the appropriate sections before making your selection.

☐ Life sciences ☒ Behavioural & social sciences ☐ Ecological, evolutionary & environmental sciences

For a reference copy of the document with all sections, see nature.com/documents/nr-reporting-summary-flat.pdf

Behavioural & social sciences study design

All studies must disclose on these points even when the disclosure is negative.

Study description	This quantitative meta-analysis includes both cross-sectional and longitudinal cohort studies.
Research sample	This review includes children under 18 years of age and reported on at least two neurodevelopmental conditions listed within the DSM-5 and assessed with reliable diagnostic measures. We searched all studies available on databases. These studies were chosen to inform our understanding of published data comparing different neurodevelopmental conditions.
Sampling strategy	The review included peer-reviewed studies in any language published from 1980 to the 19th of February 2024. Studies were included if they met our inclusion criteria (under 18 years of age, at least two neurodevelopmental conditions recruited in each published articles). Meta-analysis was conducted when at least 3 studies were reported in each comparison group. This was informed by the presence of underlying heterogeneity as well as sufficient number and balance of studies within subgroups, enabling cross-condition analyses within each cell.
Data collection	Researchers were not blinded to experimental condition during data extraction. In data extraction, EF measure outcomes (i.e., commissions or omissions errors in a task like Go-No-Go) reported in each comparison group were extracted as mean values and standard deviation scores at a single time point (or baseline results were extracted in the case of longitudinal studies). For studies reporting multiple measures derived from psychometric tests, experimental tasks and/or self/informant measures, each outcome measure was considered separately. Where there was missing data, efforts were made to contact authors regarding missing data by email at least once, however, no author was able to address these requests. In addition, all study authors were contacted for unpublished data to mitigate 'the file drawer effect'. One author was able to address this request and their data is included in the results.
Timing	The search of eligible studies was updated in February 2024.

Data exclusions	Five studies were removed as they contributed to data asymmetry (g>2). Comparisons between neurodevelopmental conditions were not conducted if there were less than three eligible studies. Details are outlined in the methods section and was preregistered on PROSPERO.
Non-participation	No participants were recruited in this systematic review and meta-analysis.
Randomization	Not applicable as this is a systematic review and meta-analysis study.

Reporting for specific materials, systems and methods

We require information from authors about some types of materials, experimental systems and methods used in many studies. Here, indicate whether each material, system or method listed is relevant to your study. If you are not sure if a list item applies to your research, read the appropriate section before selecting a response.

Materials & experimental systems

n/a	Involved in the study	
☒	☐	Antibodies
☒	☐	Eukaryotic cell lines
☒	☐	Palaeontology and archaeology
☒	☐	Animals and other organisms
☒	☐	Clinical data
☒	☐	Dual use research of concern
☒	☐	Plants

Methods

n/a	Involved in the study	
☒	☐	ChIP-seq
☒	☐	Flow cytometry
☒	☐	MRI-based neuroimaging

Plants

Seed stocks	Not applicable.
Novel plant genotypes	Not applicable.
Authentication	Not applicable.
