# Supplementary material for: Executive function in children with neurodevelopmental conditions: a systematic review and meta-analysis
Source: Nat Hum Behav. 2024 Oct 18;8(12):2357–66. doi: 10.1038/s41562-024-02000-9 (PMC11659155; doi:10.1038/s41562-024-02000-9)
Supplement: Supplementary file 3 — Statistical analysis results from metafor package for Supplementary Fig. 1a,b. Supplementary Fig. 2. Effect sizes for seven areas of EF across neurodevelopmental comparisons with (a) performance only and (b) informant only measures. Mean effects and their 95% confidence intervals are presented by the central black circle and the horizontal line. The size of the black circle reflects the number of studies included in each comparison. The dashed line at g = 0 indicates no effect, while the P values show whether the effect was statistically significant. The P values reported from the random-effects models are two-sided. [file 41562_2024_2000_MOESM3_ESM.zip › SourceData/SourceData_SupplementaryFig1.rtf]

Data for Supplementary Figure 1A


Random-Effects Model (k = 114; tau^2 estimator: REML)

tau^2 (estimated amount of total heterogeneity): 0.1279 (SE = 0.0271)
tau (square root of estimated tau^2 value):      0.3576
I^2 (total heterogeneity / total variability):   68.24%
H^2 (total variability / sampling variability):  3.15

Test for Heterogeneity:
Q(df = 113) = 317.4802, p-val < .0001

Model Results:

estimate      se     zval    pval   ci.lb   ci.ub      
  0.6070  0.0427  14.2059  <.0001  0.5232  0.6907  *** 

---
Signif. codes:  0 '***' 0.001 '**' 0.01 '*' 0.05 '.' 0.1 ' ' 1


Data for Supplementary Figure 1B


Estimated number of missing studies on the left side: 0 (SE = 5.9972)

Random-Effects Model (k = 113; tau^2 estimator: REML)

tau^2 (estimated amount of total heterogeneity): 0.0936 (SE = 0.0223)
tau (square root of estimated tau^2 value):      0.3059
I^2 (total heterogeneity / total variability):   61.26%
H^2 (total variability / sampling variability):  2.58

Test for Heterogeneity:
Q(df = 112) = 269.8321, p-val < .0001

Model Results:

estimate      se     zval    pval   ci.lb   ci.ub      
  0.5860  0.0390  15.0419  <.0001  0.5096  0.6623  *** 

---
Signif. codes:  0 '***' 0.001 '**' 0.01 '*' 0.05 '.' 0.1 ' ' 1
